# Enough but not too many: A bi-threshold model for behavioral diffusion

**DOI:** 10.1093/pnasnexus/pgae428

**Published:** 2024-10-22

**Authors:** Fahimeh Alipour, Fedor Dokshin, Zeinab Maleki, Yunya Song, Pouria Ramazi

**Affiliations:** Department of Electrical and Computer Engineering, Isfahan University of Technology, Khomeyni Shahr, Daneshgah e Sanati Hwy, Isfahan PG9G+39R, Isfahan, Iran; Department of Sociology, University of Toronto, 700 University Ave, Toronto, Ontario, Canada M5G 1Z5; Department of Electrical and Computer Engineering, Isfahan University of Technology, Khomeyni Shahr, Daneshgah e Sanati Hwy, Isfahan PG9G+39R, Isfahan, Iran; School of Communication, Hong Kong Baptist University, Hereford Road, Kowloon, Hong Kong; Department of Mathematics and Statistics, Brock University, 1812 Sir Isaac Brock Way, St. Catharines, Ontario, Canada L2S 3A1

**Keywords:** social network, information diffusion, bi-threshold model, linear threshold model

## Abstract

Behavioral diffusion is commonly modeled with the linear threshold model, which assumes that individuals adopt a behavior when enough of their social contacts do so. We observe, however, that in many common empirical settings individuals also appear to abandon a behavior when too many of their close contacts exhibit it. The bi-threshold model captures this tendency by adding an upper threshold, which, when exceeded, triggers behavioral disadoption. Here we report an empirical test of the bi-threshold model. We overcome the significant challenge of estimating individuals’ heterogeneous thresholds by extending a recently introduced decision-tree based algorithm to the bi-threshold setting. Using the context of the spread of news about three different topics on social media (the Higgs boson, the Melbourne Cup horse race, and the COVID-19 vaccination campaign in China), we show that the bi-threshold model predicts user engagement with the news orders of magnitude more accurately than the linear threshold model. We show that the performance gains are due especially to the bi-threshold model’s comparative advantage in predicting behavioral decline, an important but previously overlooked stage of the behavioral diffusion cycle. Overall, the results confirm the existence of the second upper threshold in some contexts of diffusion of information and suggest that a similar mechanism may operate in other decision-making contexts.

**Significance Statement**

From vaccines to sustainable energy behaviors to fake news, understanding how behaviors spread is critical for addressing societal issues. The behavioral diffusion is traditionally explained by the linear threshold model, assuming individuals adopt behaviors when enough of their contacts do so. We suggest, however, that often individuals will also abandon a behavior when too many others exhibit it. We specify a bi-threshold model where individuals adopt the behavior when enough, but not too many others do so. We apply the model to social media user engagement with a set of three diverse topics, as identified by hashtags or search terms. The bi-threshold model outperforms the linear threshold model by orders of magnitude on predicting user engagement.

## Introduction

Social networks facilitate the spread of news, gossip, behaviors, and products. Granovetter (1) specified a simple and intuitive mechanism that underpins much of the research on social network diffusion: individuals adopt a behavior if enough others do so. More specifically, in an interacting population of individuals where a behavior is spreading, each individual has a particular “threshold” and adopts the behavior if the proportion of others who have already adopted the behavior exceeds the threshold.

Numerous studies have since used the threshold model and its extensions to explain spreading phenomena (2–5). Across diverse domains, including misinformation (6), health behaviors (7), and innovation (8), threshold models have been shown to explain adoption dynamics. However, adoption booms are commonly followed by periods of bust—riots come to an end, new fashions go out of style (9–11), and juicy gossip turns to stale news. Yet threshold models of diffusion have surprisingly little to say about this common stage of diffusion.

**Fig. 1.**
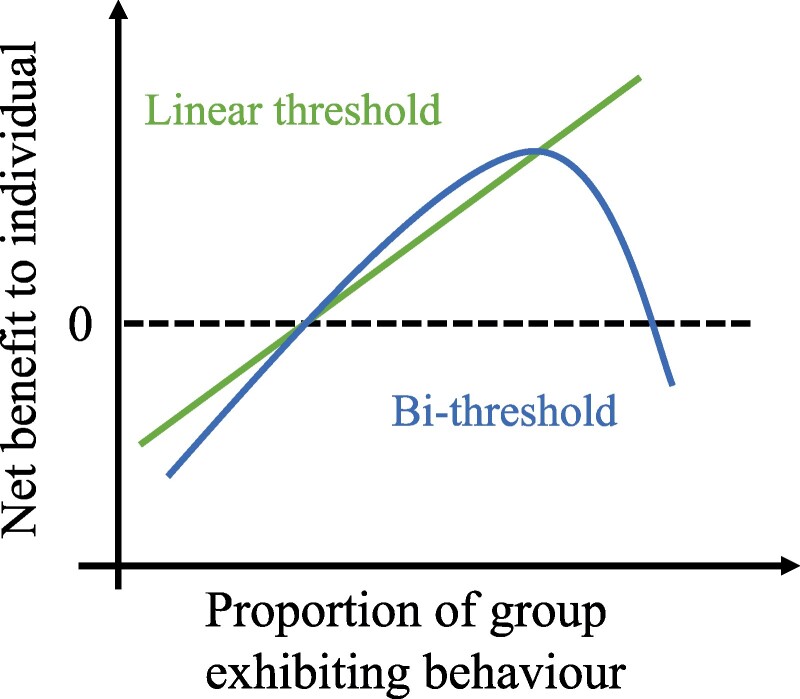
Stylistic examples of the net-benefit curve for the linear threshold model (straight line, green) and the bi-threshold model (curved line, blue).

We suggest that, in many important cases, such declines can be explained endogenously by a specific structure of individual preferences. *Linear threshold models*, which Granovetter introduced and most studies of diffusion still rely upon, assume that the net benefit to individuals strictly increases with the number of previous adopters. This is a reasonable assumption in some cases, for example when the diffusion behavior or product has strong network externalities (12), but there are many important contexts where we should anticipate a deceleration and an eventual decrease of the net benefit with increasing adoption among network neighbors. This inverted-U shape of the net benefit curve, visualized in Figure 1 implies the existence of an upper threshold, at which the individual may choose to disengage in the behavior, abandon the product, or quit spreading the rumor. Previous work (13, 14), including by Granovetter (1, 15), has hinted at the theoretical importance of a possible second threshold, but it has not been explored in detail and never tested empirically.

We identify three distinct mechanisms elaborated in previous social science research that imply the *bi-threshold model*, composed of a lower threshold that initiates adoption and an upper threshold that initiates disadoption. First, negative network externalities (or congestion effects) are a common feature of many nonexcludable goods (16, 17). For example, a beach day with a couple of friends may entice one to join, but a crowded beach will send one packing. The second mechanism is distinction (or the snob effect) (18, 19), which suggests that in many domains, but especially when it comes to cultural tastes, individuals have a substantial desire to stand out from the crowd. A fashion-conscious person may adopt a style after encountering it a few times, but will retire it when it becomes too common. The third mechanism is saturation. We refer to saturation when the net benefit of persisting with a behavior depends on the existence of nonadopters. This may be especially common in the case of informational diffusion, in which continued dissemination of gossip, a news item, or a joke loses its appeal with more people being in the know. The dynamic is captured by common idioms like “beating a dead horse” and “preaching to the choir.”

The lack of empirical evidence for the bi-threshold model is due in part to the challenge of estimating heterogeneous thresholds empirically. Even in the case of the simpler, single-threshold model, researchers tend to avoid estimating heterogeneous thresholds from data, assuming instead a single homogeneous threshold (20), random thresholds (21), or thresholds that are a simple function of node degree (22). Our approach builds on a recent advancement in estimating linear thresholds by Tran et al. (23, 24). Tran et al. proposed a tree-based learning algorithm (Causal Tree Learning or CTL) for estimating linear thresholds, which assumes that individuals with the same features have the same threshold and this threshold maximizes the conditional average treatment effect (CATE), i.e. the social influence difference between when they are active and inactive. We extend this algorithm to bi-threshold models, which enables us to simultaneously estimate heterogeneous lower- and upper-thresholds from empirical data.

We test the bi-threshold model in the context of information diffusion on social media. Our primary case study traces the spread of the news about the discovery of the Higgs boson between July 1, 2012 and July 8, 2012 on Twitter. We also replicate the analysis on two additional datasets, the spread of discussion about the COVID-19 vaccination campaign on Weibo (a Chinese-language social media platform) and the spread of discussion of the Melbourne Cup, a popular horse racing event in Australia. See Materials and Methods for details about each dataset. Due to space considerations, we focus on the results from the Higgs boson dataset here and present the results for the additional datasets in the Supplementary material.

Social media is a strategic and important setting for research on behavioral diffusion. One key advantage is that digital trace data from social media allow us to meet the substantial data demands for the model estimation task. Specifically, we require a longitudinal dataset of a behavior spreading in a population, including a measure of each individual’s behavior and social contacts at every time period. Additionally, information diffusion is an issue of critical social importance, especially in the age of social media, where useful information and misinformation compete for attention. Diversity of the diffusing topics in our analyses as well as the use of two alternative social media platforms provides more confidence about the robustness of our empirical test and its generalizability.

The study asks the question: under what conditions do individuals spread information about a new topic? The dominant, linear threshold model holds that individuals will pass on the information when they witness a sufficient number of neighbors doing so. The bi-threshold alternative adds an important caveat: individuals will spread information when enough others are doing so, but will disengage when an upper threshold is reached. Empirically, we use the CTL method and extend it to the bi-threshold case to train linear and bi-threshold models on empirical data of the spread of news about the Higgs boson (or COVID-19 vaccination or the Melbourne Cup). We then compare the performance of the two models in predicting future engagement with the news. Across all three datasets, we find that the bi-threshold model outperforms the linear threshold model in the prediction task and show that the bi-threshold model’s performance advantage can be attributed to its enhanced ability to capture dynamics of behavioral decline.

## Model

Consider a social network of individuals that decide whether to share a piece of information at times t=1,2,…. Individual *i* is connected to individual *j* by an edge of weight $w_{ij}$, where $w_{ij}$ captures the amount of influence. At each time *t*, conditional on being active or “logged in” (see below for details), the individual makes a decision, denoted by $y_{i}$, which equals 1 if she shares information and 0 otherwise. For example, in the Twitter network, individuals represent users, individual *i* is linked to *j* if she “follows” *j* and shares information by reacting to a tweet with a certain hashtag by say retweeting it. The linear (single) threshold model assumes a time-invariant threshold $\theta_{i}\in [0,1]$ for every individual *i* and implies that individual *i*, active at time t, decides to diffuse information at time t+1 if the influence of her neighbors exceeds her threshold:


(1) $$y_{i}(t+1)=\left\{ \begin{array}{ll} 1, & \sum_{\;j\in N_{i}}w_{ij}y_{\;j}(t)\geq \theta_{i},\;\\ 0, & \text{otherwise}, \end{array}\right.$$

where $N_{i}$ is the set of individuals that *i* is linked to. The bi-threshold model, on the other hand, assumes a lower and an upper threshold $\theta_{i}^{\mathrm{lower}},\theta_{i}^{\mathrm{upper}}\in [0,1]$ for every individual *i* and implies that individual *i*, active at time *t*, diffuses information at time t+1 if the influence of her neighbors exceeds her lower threshold but falls short of her upper threshold:


(2) $$y_{i}(t+1)=\left\{ \begin{array}{ll} 1, & \theta_{i}^{\mathrm{lower}}\leq \sum_{\;j\in N_{i}}w_{ij}y_{\;j}(t)\leq \theta_{i}^{\mathrm{upper}},\;\\ 0, & \text{otherwise}. \end{array}\right.$$

**Fig. 2.**
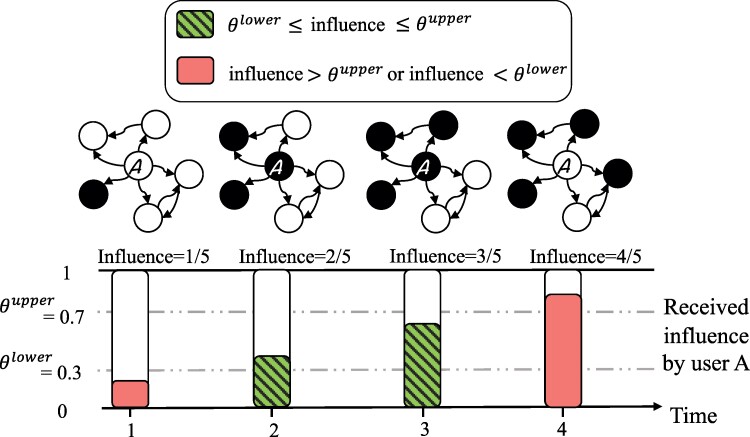
Illustration of information diffusion on social media under the bi-threshold model. User *A* follows five other users (indicated by arrows in the network graphs). Users are colored black when they are engaged with the spreading news and white otherwise. The focal user *A* has a lower-threshold equal to 0.3 and an upper-threshold equal to 0.7. The bars are placed at four time-steps, with height corresponding to influence received by user *A* and color indicating whether the user is engaged with the news (green) or not (pink). User A has a lower-threshold of 0.3 and an upper-threshold of 0.7. The user is not engaging with the news at t=1, when just 1/5=0.2 of her neighbors has engaged with the news. User A begins to engage with news at time 2, when a second neighbor engages with it and the lower threshold is surpassed (2/5>0.3). Finally, user A stops engaging with the news when four of her five neighbors engage with it, surpassing the upper threshold value (4/5>0.7).

Figure 2 illustrates the diffusion process under the bi-threshold model.

Fitting the models in [1] and [2] to empirical data requires two additional assumptions. First, the illustration in Figure 2 implies that users actually observe every message sent by their network neighbors. This is not necessarily true, however. Messages that drop low on a user’s message feed, whether due to overall volume of messages or time between user logins, may not be seen. In communications research this is related to the affordances of a technology, the features of a platform that interact with user perceptions and preferences to enable and constrain particular user behavior (25). We suspect that the news feed constrains the users’ ability to encounter messages that are too old, and we attempt to account for this in our modeling by introducing a parameter ($T_{\mathrm{effect}}$), which captures the length of interval that a neighbor’s message will be seen by the focal user. The baseline specification, reported below, assumes a $T_{\mathrm{effect}}$ value of ∞, but we show that alternative specifications do not affect our findings Supplementary Material, [14]. Second, to generate model predictions, we must specify how often users have the opportunity to take an action (e.g. tweet). Patterns that assume log-ins that are more frequent than is empirically the case, will systematically overestimate the overall volume of user engagement. We thus specify a parameter ($T_{\mathrm{login}}$), representing the set of times when a user may be expected to participate. We tested intervals of different length and also modeled intervals as a function of time since last documented activity. We found that a pattern representing one login per 24 hours provides the best fit to the data and we use this specification in the results presented below. See Materials and Methods for full details about the implementation of $T_{\mathrm{effect}}$ and $T_{\mathrm{login}}$, Supplementary Material, [15] for an illustration of how the two parameters are integrated into the model, and Supplementary Material, [14] for model comparisons across a wide range of $T_{\mathrm{effect}}$ and $T_{\mathrm{login}}$ values, which documents the robustness of the results.

Our empirical analysis compares the performance of the linear and bi-threshold models for predicting the aggregate number of social reactions to the Higgs boson news on Twitter, the COVID-19 vaccination campaign on Weibo, and the Melbourne Cup on Twitter. We extend the CTL method to the bi-threshold model to estimate thresholds for our population of social media users from the training dataset (i.e. all time intervals preceding the testing intervals, described next). We then use the trained models to generate estimates for the subsequent four 6-hour intervals (the testing dataset). By shifting the testing data one 6-hour interval at a time, we generate 19 estimates of aggregate activity across seven days of the news spreading episode (2012 July 2–2012 July 7). The first five time periods are used to train the model and the final four periods are excluded because of insufficient test intervals remaining. We replicate the same analysis for the Weibo-COVID and the Mel-Cup datasets and present these results in Figures S19–S24.

## Results

**Fig. 3.**
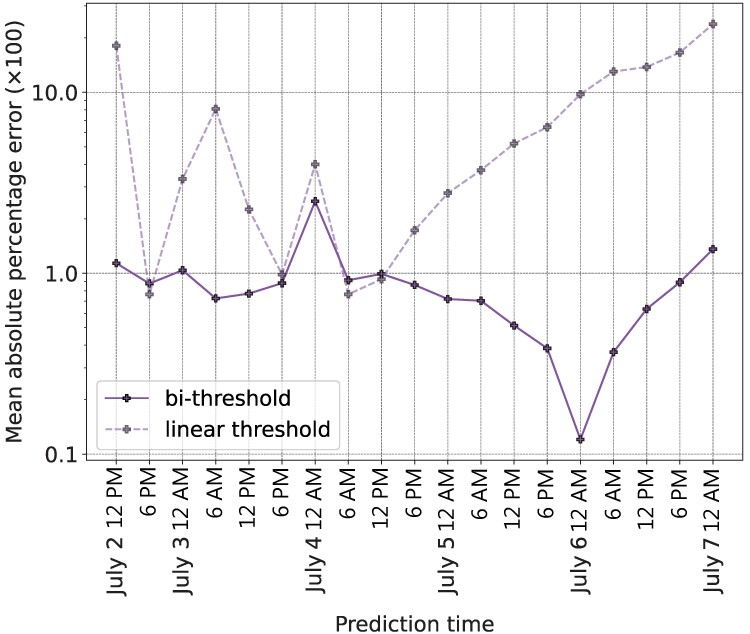
Performance comparison of the linear threshold and bi-threshold models without an effective time and for a login frequency of 24 hours. Each point represents the mean absolute percentage error of predicting the future 6, 12, 18, and 24-hour number of tweets, retweets, and replies to the Higgs boson news hashtags. For the first point (July 2, 12 PM) both models were trained using the data prior to that time, i.e. July 1, 5 AM to July 2, 11 AM, and were tested at the points July 2, 12 AM, July 2, 6 PM, July 3, 12 AM, and July 3, 6 AM. For each subsequent point on the *x*-axis, the process was repeated but the previous time periods were added to the training dataset. The individuals’ thresholds were estimated using the training dataset and then used to make predictions for the four time periods in the testing dataset. The original linear and bi-threshold models [1] and [2] were used without the login and effective time compartments, i.e. $T_{\mathrm{login}}=24$ and $T_{\mathrm{effect}}=\infty$. The bi-threshold model outperforms the linear threshold model at every point in the testing dataset.

Figure 3 presents results comparing the performance of the linear and bi-threshold models in predicting the volume of Twitter activity about the Higgs boson during the 19 test periods. We measure performance as the mean absolute percentage error of each model’s prediction of the number of social reactions during each test period. The bi-threshold model outperformed the linear-threshold model overall and at 16 of 19 time periods. The linear threshold model performed particularly poorly in the second half of the diffusion episode, after engagement with the news reached its peak. These results are based on the baseline specification of the linear-threshold and bi-threshold models, which assumes $T_{\mathrm{effect}}=\infty$, representing the case in which all network neighbor’s tweets and social reactions remain observable indefinitely.

**Fig. 4.**
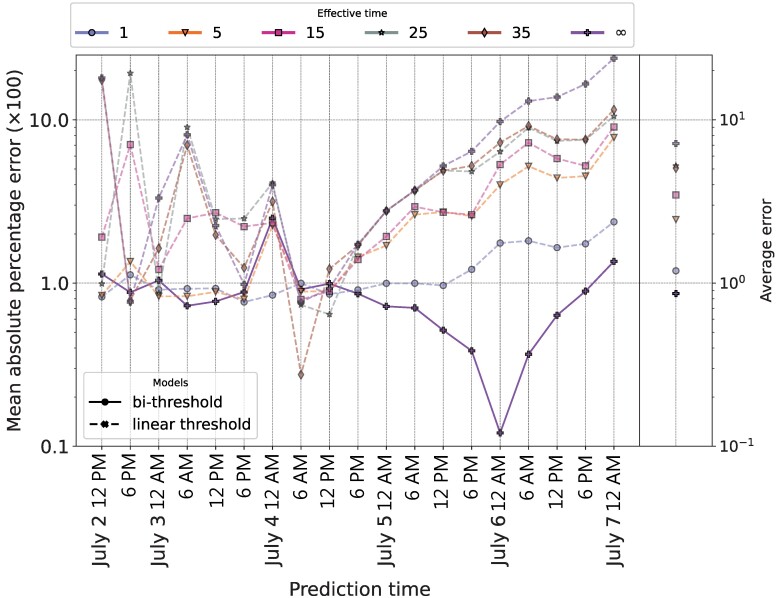
Performance comparison between the optimized bi-threshold model and the top-performing linear-threshold models taking a range of $T_{\mathrm{effect}}$ values. The lines on the left-hand side of the figure track the Mean absolute percentage error across the 19 test intervals, while the dots in the right-hand panel represent the average error rate across the entire diffusion episode. The optimized bi-threshold model has $T_{\mathrm{login}}=24$ and $T_{\mathrm{effect}}=\infty$. All linear threshold models have $T_{\mathrm{login}}=24$, an optimal value across model types, while $T_{\mathrm{effect}}$ varies from 1 to ∞.

To ensure that the results are not driven by this assumption, we test the performance of the linear threshold and bi-threshold models across a wide range of $T_{\mathrm{effect}}$ values and present the results in Figure 4. The bi-threshold model outperformed every specification of the linear threshold model, achieving the lowest overall average error. Across the specifications, the bi-threshold model had a distinct performance advantage during the decline of activity from its peak. Supplementary Material, [14] presents results from the full robustness analysis, including from models with alternative specifications of $T_{\mathrm{login}}$.

**Fig. 5.**
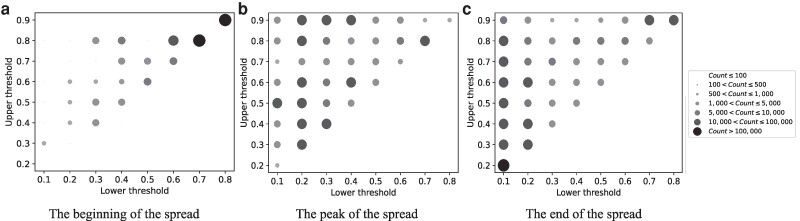
Distribution of threshold estimates across different stages of the Higgs boson news spread. The distribution of the estimated lower and upper thresholds for the bi-threshold model with $T_{\mathrm{effect}}=\infty ,T_{\mathrm{login}}=24$ are provided for the time intervals a) July 2, 12 AM to July 3, 6 PM, as the beginning of the news spread, b) July 4, 12 AM to July 5, 12 AM, as the peak of spread, and c) after July 5, 6 AM, as the final stage of the spread. The size of the circles is proportional to the frequency of individuals with the specified lower and upper thresholds.

Finally, in Figure 5, we present the distributions of the estimated thresholds for the bi-threshold model across three time periods, (a) beginning of the spread (July 2, 12 PM to July 3, 6 PM), (b) peak of the spread (July 4, 12 AM to July 5, 12 AM), and (c) end of the spread (July 5, 6 AM to July 7, 12 AM). We observe that estimated lower and upper thresholds varied significantly over time and their values help to explain the model’s comparative advantage. During the beginning of the spread most individuals had very high lower thresholds, which constrained the number of individuals who engaged with the news in the initial phase. During the peak of the spread, we observe significant density in the upper left region of the plot where lower thresholds are low and upper thresholds are medium or high. This combination permits behavioral engagement under a wide range of local contexts. Finally, toward the end of the spread, many individuals continue to have low lower thresholds, but upper thresholds also decrease on average, thus imposing a limit on the spread. In the Figures S2 to S13 provide further insight into the model’s performance by reporting the different states that users are predicted to occupy over time, i.e. for the bi-threshold model, whether users are active or inactive, and whether inactive users are offline, do not have enough contacts promoting the news (below their lower threshold), or have too many contacts promoting the news (above their threshold). The graphs illustrate that the bi-threshold model has a distinct advantage over linear threshold specifications by allowing users to become inactive after their upper threshold is reached and thus identifying as active users only those whose local contexts have not yet been saturated with the spreading news.

## Discussion

The linear threshold model has been singularly influential in diffusion research, holding that individuals adopt a behavior if enough others do so. Our results suggest an important corrective to the linear threshold paradigm, at least in some contexts: individuals adopt a behavior if enough—but not too many—others do so. Our empirical study compared the performance of the bi-threshold and linear models for predicting the spread of news on Twitter. The bi-threshold model outperformed the linear threshold model in the prediction task across a wide range of possible specifications, with a particular advantage in performance during the decline phase of the diffusion episode.

The bi-threshold model provides analytic traction for the many contexts where an individual’s motivation to adopt a behavior is a concave function of the behavior’s current prevalence. Our results show that the bi-threshold model performs about as well as the linear threshold model for predicting a behavior’s growth, and has a distinct relative advantage in predicting behavioral decline, an area that has previously been identified as a blind spot in diffusion research (26, 27). To the extent previous research has examined behavioral disengagement, it has tended to specify it as an anticoordinating tendency among a subset of actors, i.e. as a unique preference for distinction that stands in contrasts with the prevailing preference for conformity among the rest of the population. Our results question the validity of a hard separation between conforming and anticoordinating actors. In many important instances of diffusion, a more accurate explanation of individual behavior may emerge if we assume that individuals transition between conformity and distinction based on the overall prevalence of the behavior in the population.

We observe that our bi-threshold model differs from the one proposed in (28). In their model, which the authors applied to the study of smoking behavior, individuals also have two thresholds. However, in their model, an individual initiates the behavior when one threshold is passed (e.g. a sufficient number of smoking friends influence the nonsmoker to start) and desists only when a second, lower threshold is met (e.g. a sufficient number of the smoking friends become nonsmokers). This is very different from our formulation of a lower and upper threshold.

Our analysis also supports the hypothesis that individuals’ thresholds change over time. In the case of the spread of news about the Higgs boson on Twitter, most individuals displayed a reluctance to engage with the news as their lower thresholds were high. The news gained a foothold among a small segment of the population that had relatively lower values of lower thresholds during this initial period. The news then spread rapidly and, at the peak, many individuals had low lower thresholds and high upper thresholds, allowing for behavioral engagement under a wide range of local contexts. During the decline of the behavioral spread, however, the upper threshold shifted to lower values, thereby slowing the spread of the news. A small subset of individuals continued to have a relatively high upper threshold even during this period of decline, thus sustaining a low level of activity. We note that the best fitting bi-threshold model had $T_{\mathrm{effect}}=\infty$, implying that users observed (and were influenced by) all of their neighbors’ previous engagements with the news during the full diffusion episode, which lasted about a week. Thus, the estimated thresholds reflected the accumulation of peers’ social reactions across the full week.

The estimated threshold distribution also suggests the importance of relaxing the homogeneous threshold assumption for modeling the lifecycle of behavioral spread. In the earliest stage of diffusion, agents are most similar on their threshold values. Yet even here, the news gains a foothold by virtue of a small subset of actors with lower values of lower thresholds. As the diffusion process unfolds, lower and upper thresholds become more heterogeneous. At peak and at the end of the spread, the population is characterized by a variety of threshold values.

The present study’s primary contribution is theoretical, but we also advance empirical approaches to studying diffusion. First, we adapt recent developments in approaches to estimating heterogeneous thresholds (the CTL method) to the context of the bi-threshold model. Second, our parameterization of $T_{\mathrm{effect}}$ and $T_{\mathrm{login}}$ provide a new framework for using social media data in the empirical study of diffusion. These parameters allow us to be more sensitive to alternative affordances of social media technologies, which affect how often users are available to engage and users’ ability to observe messages from their network (25). More generally, $T_{\mathrm{effect}}$ and $T_{\mathrm{login}}$ make explicit the importance of temporal alignment for transmission of a social contagion. In other words, network ties do not automatically conduct all available information; rather, transmission depends on the coincidence of the adopter’s actions and the potential adopter’s observation of those actions. This issue can also be recast as one of network measurement: any static recording of a social network simplifies the real-world structure of social influence, which has a significant stochastic component (29). Our two parameters do not fully capture this richness, but they make the problem explicit and take an incremental step toward addressing it in empirical studies of diffusion.

Despite these advancements, the study also has important limitations that could be addressed in future research. We had access to only a limited set of features about individual users, constructed from users’ recorded activities and social network connectivity. These features are key inputs for the CTL method of estimating heterogeneous thresholds and a richer feature set would have likely provided better estimates. Finally, our empirical tests of the bi-threshold model focused on information diffusion on social media. This choice of research setting was guided by the availability of data required to fit these models, and we sought to provide some assurance of robustness by replicating the analysis on three diverse topics sourced from two different social media platforms (Twitter [or X] and Weibo). We thus make an important contribution to the theory of social contagion by demonstrating that the bi-threshold model provides a better fit to the data in this set of cases. Additional research is needed, however, to flesh out the scope conditions for the bi-threshold model. We proposed three mechanisms under which we expect an upper threshold to be important–congestion effects, snob effects, and saturation. Future work should build on our initial results to test these mechanisms directly, including by developing new empirical strategies for studying diffusion in more diverse contexts and in offline settings.

## Methods

### Data

The primary case study focuses on the spread of the news about the Higgs boson discovery on Twitter (now called X), collected for the period between 2012 July 1 and 2012 July 8 (30). The dataset contains all tweets that included one of the following hashtags: #LHC, #Higgs, #CERN, and #boson. It captures the activity of 456,626 Twitter users, who have 14,855,842 following relationship edges among them. We focus attention on the “tweet,” “retweet,” and “reply” social reactions, and exclude “likes” and “mentions” from the analysis. Two additional datasets were used to replicate our analyses. The first dataset is also from Twitter and contains all tweets that included one of the following terms: “Melbourne Cup,” “#MelCup,” and “DerbyDay, from 2017 November 6 to 2017 November 8. The resulting dataset captures the activity of 1,437 Twitter users, who had 70,806 following relationships among them. The second replication dataset is from Weibo, a popular Chinese-language messaging platform. The dataset contains all messages posted between 2021 April 29 and 2021 May 18 that included one of the following terms in Chinese: “vaccine,” “vaccination,” “adenovirus vector,” “inactivation,”“clinical trail,” “Phase III trail,” “immune,” “antibody,” “mutant virus,” “herd immunity,” “novel coronavirus,” “corona virus,” “COVID,” “novel corona pneumonia,” “Wuhan’s unknown pneumonia,” “pneumonia of unknown cause,” “nucleic acid testing,” “Wuhan pneumonia,” “human-to-human transmission,” and the following in English: “COVID-19,” “COVID,” “COVID19,” “SARS,” “SARS-2,” “SARS-CoV-2,” “nCoV,” “2019-nCoV.” It captures the activity of 1,052,896 users, who have 3,841,030 following relationships among them. We focus attention on the “post,” “re-post,” and “comment” social reactions.

We transformed the raw datasets into three hourly datasets, creating a binary indicator of each user *i*’s decision ($y_{i}(t)$) to engage at time *t*, which equals 1 if the user recorded a social reaction to any message with a relevant term at least once during that hour and 0 otherwise. We assumed a uniform distribution of edge weights among a user’s followees, i.e. $w_{ij}=1/|N_{i}|$ for all $j\in N_{i}$. We then calculate the social influence received by each user at time *t* based on the activity in the user’s social network and conditioned by specific values of login and effective time (see below).

### Model specification

[1] and [2] above show the specifications of the linear threshold and bi-threshold models, respectively. These two models assume that (i) individuals are always available to engage with a tweet and (ii) individuals observe all of their neighbors’ tweets. We extend these models to relax each of these assumption. Since users may not be active on Twitter at all times, we specify *login time* to reflect the time periods when a user *could* decide to engage. We consider three different login patterns. First, we consider a *constant* duration of time between two consecutive activations (log-ins) of every individual, denoted by $T_{\mathrm{login}}$. For example, if $T_{\mathrm{login}}=5$, the users log in every 5 hours. The second log-in pattern is the same as the first with the caveat that after an individual becomes active, they stay more active for the next period, with the frequency of logins decreasing linearly back to the constant $T_{\mathrm{login}}$ value. For example, if $T_{\mathrm{login}}=5$ and the user retweets a message at t=10, then the user’s log-in times would be


t=0,5,10,11,13,16,20,25,30,35,….

The process is reset if the user retweets at any of the above time steps, e.g. if the user also retweets at time 13, then the log-in times become:


t=0,5,10,11,13,14,16,19,23,28,33,38,….

A final wake-up pattern we consider has the same structure as the previous one, but with an exponential rather than linear decay, i.e. the next log in after a retweet would happen an hour later, then 2, then 4, until the original $T_{\mathrm{login}}$ is reached. With $T_{\mathrm{login}}=5$ and a retweet at t=10, the log-in pattern would be


t=0,5,10,11,13,17,22,27,32,37,….

We relax the assumption that individuals observe all the neighbors’ tweets by defining effective time, $T_{\mathrm{effect}}$, as the duration that the influence of a network neighbor’s activity can be seen by the focal user (i.e. before falling too far down in the feed to be noticed). We test many values of $T_{\mathrm{effect}}$, ranging from 1 to ∞. After incorporating the effective and login times, the bi-threshold model [2] becomes


(3) $$y_{i}(t+1)=\left\{ \begin{array}{ll} 1, & \theta_{i}^{\mathrm{lower}}\leq \sum_{\;j\in N_{i}\cap E(t)}w_{ij}\leq \theta_{i}^{\mathrm{upper}}\;\text{and}\;i\in A(t)\;\\ 0, & \text{otherwise}, \end{array}\right.$$

where E(t) is the set of individuals whose influence are still effective at time *t*, and A(t) is the set of active individuals at time *t*. The modified version of the linear threshold [1] can be obtained similarly.

### Model estimation

To estimate heterogeneous thresholds for the users based on their characteristics (features) *X*, over the training dataset, we extended CTL model (23), which is for estimating a single linear threshold for each user. In the linear threshold case, the single threshold for users with same features X=x is obtained as the $\theta_{x}$ that maximizes the following term:


(4) $$E_{i\in A(t),t}\left[y_{i}(s_{i}(t)\geq \theta_{x})-y_{i}(s_{i}(t)<\theta_{x})|X=x\right]$$

where $s_{i}(t)$ is the social influence received by user *i* at time *t* and equals $\sum_{\;j\in N_{i}\cap E(t)}w_{ij}(t)$ as in [3]. The term $y_{i}(s_{i}(t)\geq \theta_{x})$ is the action of user *i* when her received social influence exceeds her (estimated) threshold whereas $y_{i}(s_{i}(t)<\theta_{x})$ is her action when her received influence falls short of the threshold. Ideally, the threshold $\theta_{x}$ should be estimated to make the first term one and the second zero, resulting in a difference of positive one, which is the maximum possible difference. Poor estimates of the threshold, on the other hand, may result in a negative difference. By maximizing the expectation of the difference over all active users, i∈A(t) over all hours *t* in the training dataset, the optimal threshold based on the given features *x* is obtained.

Another optimization is needed over the features *x* to obtain the optimal categorization of the users that would consequently lead to the optimal threshold for each category. A tree-based searching algorithm was used in (23) to find the optimal categories. First, the users are divided into two sub-populations based on a single feature, such that a certain *partition measure* based on [4] is maximized over the two sub-populations. The same process is repeated for each sub-population in the subsequent levels of the tree, until further partitioning does not increase the partition measure. The estimated threshold at each leaf of the tree then is the optimal threshold for the sub-population with the features specified over the levels of the tree. To increase the performance of the optimization algorithm, the training dataset is further partitioned into a training and validation dataset and the partition measure is defined accordingly. We skip the details here and refer the reader to (23).

To estimate the upper- and lower-thresholds of the bi-threshold model, we extended the difference term in [4] as follows:


(5) $$y_{i}\left(s_{i}(t)\in (\theta_{x}^{\mathrm{lower}},\theta_{x}^{\mathrm{upper}})\right)-y_{i}\left(s_{i}(t)\notin (\theta_{x}^{\mathrm{lower}},\theta_{x}^{\mathrm{upper}})\right).$$

As with the potential features, we used the users’ out-degree and in-degree from the Twitter follower graph, as well as their number of retweets, reply, neighbors’ retweet, and neighbors’ replies from the time interval in the training dataset.

### Model evaluation

To evaluate model performance, we specified training and test datasets as follows. For the Higgs boson data, we created 6-hour time steps from the hourly dataset, starting on July 2, 12 PM and ending July 7, 12 AM. We then used each model to predict the number of active users in the 6-hour time steps, four at a time. Specifically, the test datasets were 24-hour intervals [July 2, 1 PM–July 3, 12 PM], [July 2, 7 PM–July 3, 6 PM], …, [July 7, 1 AM–July 8, 12 AM] that we shifted successively by 6 hours. Each interval, e.g. [July 2, 7 PM–July 3, 6 PM] was divided into four 6-hour intervals: [July 2, 7 PM–July 3, 12 AM], [July 3, 1 AM–July 3, 6 AM], [July 3, 7 AM–July 3, 12 PM], and [July 3, 1 PM–July 3, 6 PM]. For each 6-hour interval, we considered all users who became active at least once during the interval as an active user, resulting in a single prediction, say $\hat{n}$. By comparing this prediction to the true number of active users in that interval, denoted by *n*, we compute the mean absolute percentage error for the interval, i.e. $100|\hat{n}-n|/n$, and then average the four errors over the four 6-hour time steps. The data prior to each interval was used for training the model. Note that this means that early periods had much less training data available compared to later time periods. In the Supplementary material, we provide two alternative evaluation designs. Figure S17 reports the accuracy of model predictions when they are extended to the end of the diffusion period, rather than just the following 24 hours. Figure S25 adjusts the training dataset to be of equal size throughout the analysis, thus reducing the absolute accuracy of the model in later period, but making the predictions more comparable across the diffusion period. Results for these alternative evaluations are consistent with those reported in the paper.

For the Melbourne Cup dataset, the time interval from 1 PM 6 November 2017 to 6 PM 6 November 2017 was used only for training, and the time interval from 6 PM 7 November 2017 to 4 AM 8 November 2017 was used only for testing the models. The interval from 6 PM 6 November 2017 to 6 PM 7 November 2017 was used for both training and testing in 6-hour time steps, similar to the approach used for the Higgs boson dataset. Finally, because the Weibo-COVID dataset had much sparser activity, a larger interval, from 12 AM 29 April 2021 to 12 PM 16 May 2021, was used for training. Smaller training datasets provided insufficient information for learning and produced poor estimates. The interval from 12 AM 16 May 2021 to 4 AM 18 May 2021 was used for both training and testing.

## Supplementary Material

pgae428_Supplementary_Data

## Data Availability

The Higgs boson data used for this paper are from (30). The Melbourne Cup data are from (31). The Weibo-COVID data are collected by the authors and are available at the Zenodo repository (32).
