# Supplementary material for: Enough but not too many: A bi-threshold model for behavioral diffusion
Source: PNAS Nexus. 2024 Oct 22;3(10):pgae428. doi: 10.1093/pnasnexus/pgae428 (PMC11495329; doi:10.1093/pnasnexus/pgae428)
Supplement: pgae428_Supplementary_Data [file pgae428_supplementary_data.pdf]

Supporting Information Text

1. The number of active nodes in real dataset

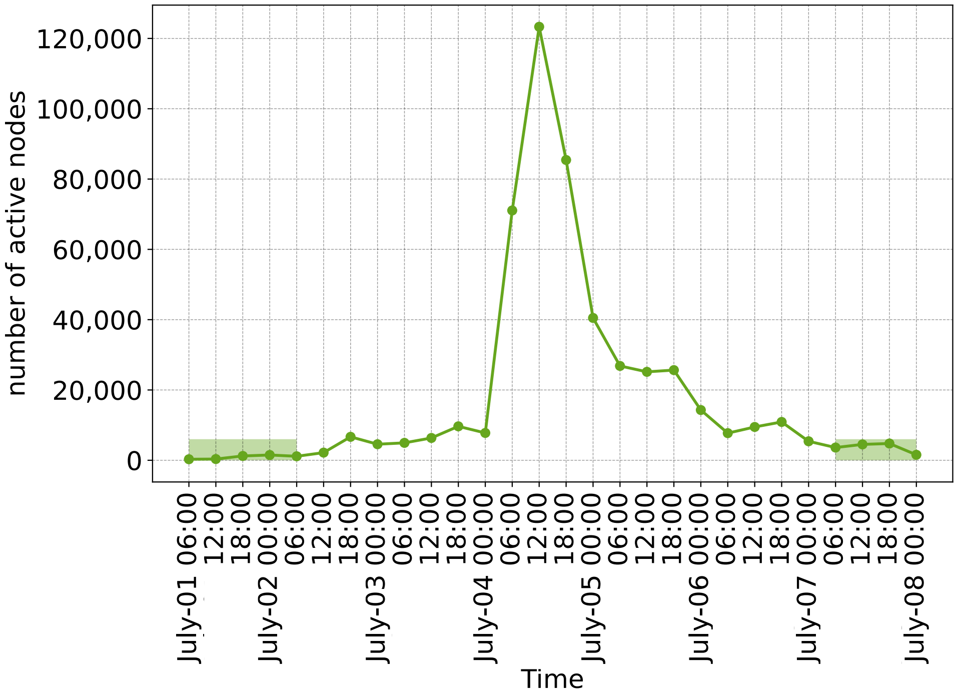

Fig. S1. The number of activity of twitter users during the spread of Higgs boson rumor. Each point represents the number of active users, whose activities include tweet, retweet and reply. We do not consider the highlighted line.

2. The estimated hourly distribution of individuals whose received social influences fall below, between, or above the lower or upper thresholds

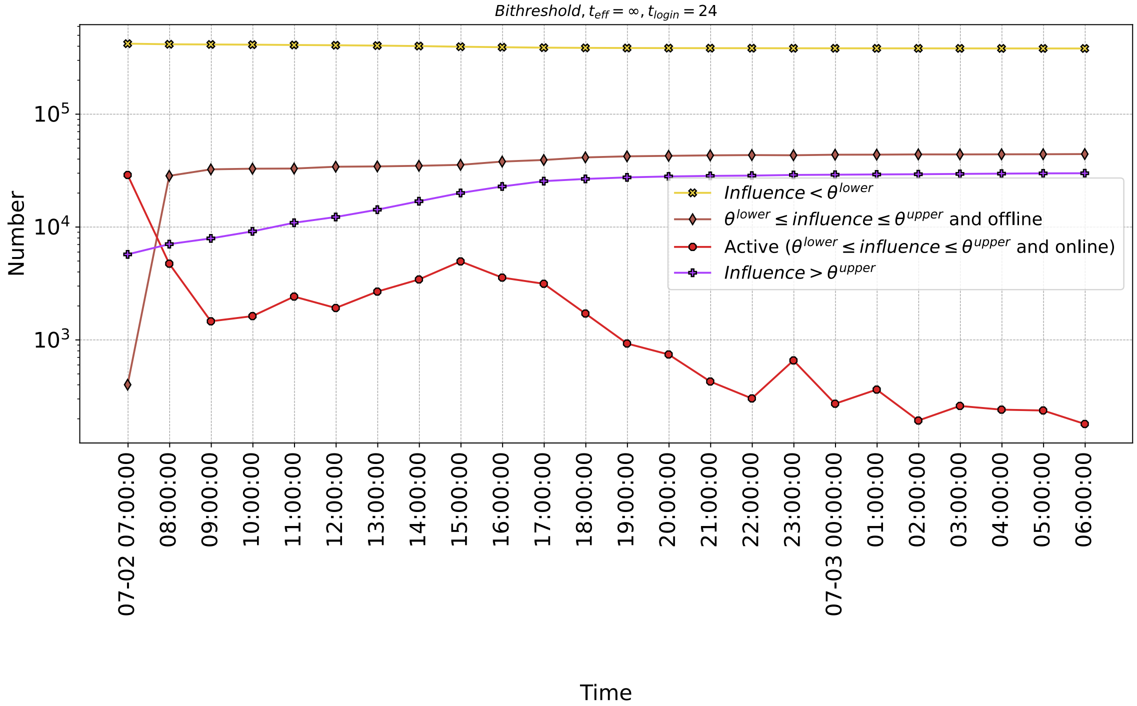

Fig. S2. Distribution of active and online, active and offline, inactive-by-the-lower-threshold, and inactive-by-the-upper-threshold users under the bithreshold model with $T_{\text{effect}} = \infty$.

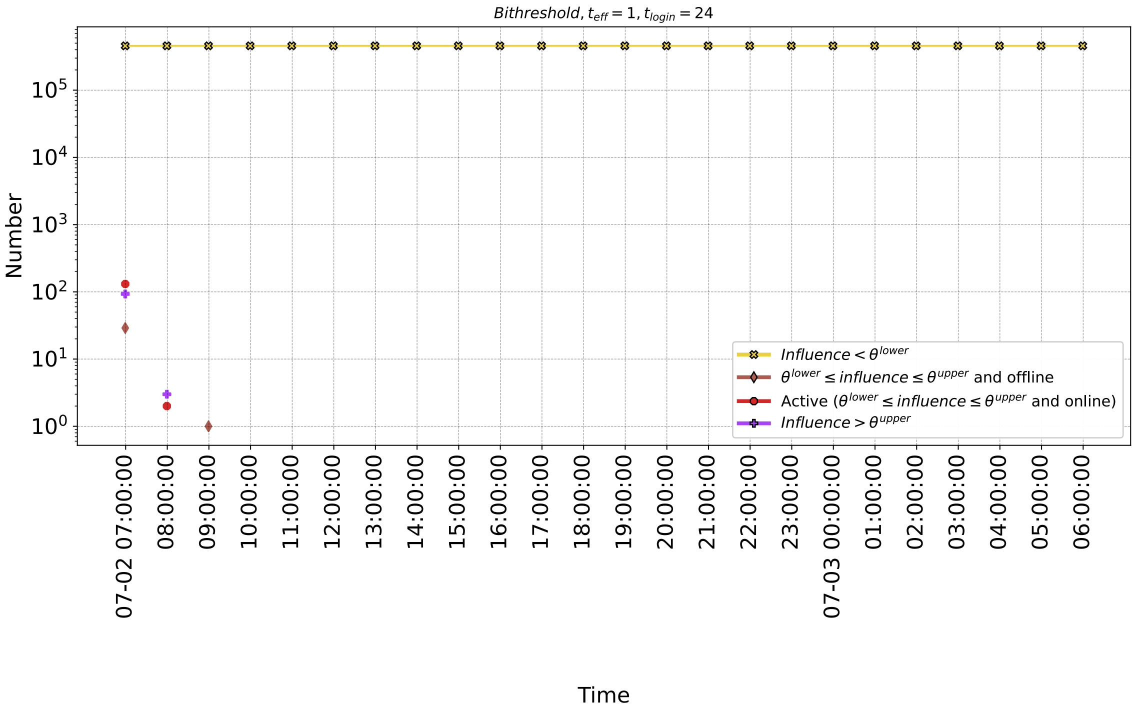

Fig. S3. Distribution of active and online, active and offline, inactive-by-the-lower-threshold, and inactive-by-the-upper-threshold users under the bithreshold model with $T_{\text{effect}} = 1$.

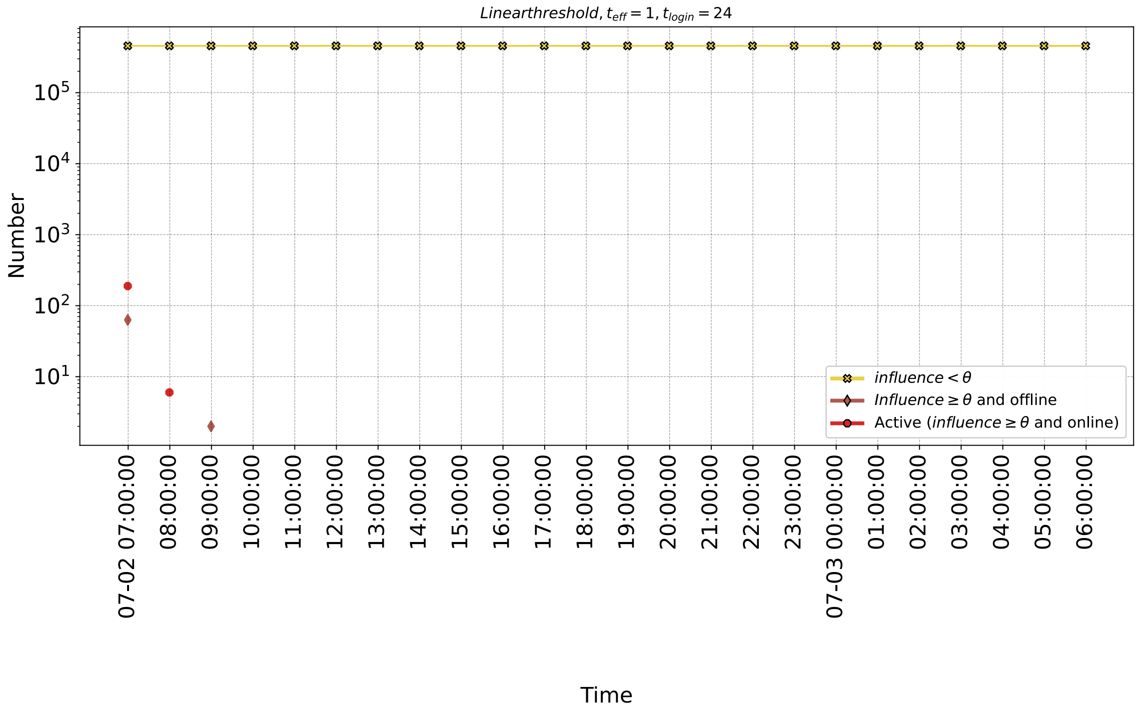

Fig. S4. Distribution of active and online, active and offline, inactive-by-the-lower-threshold, and inactive-by-the-upper-threshold users in the Higgs Boson dataset under the linear threshold model with $T_{\text{effect}} = 1$.

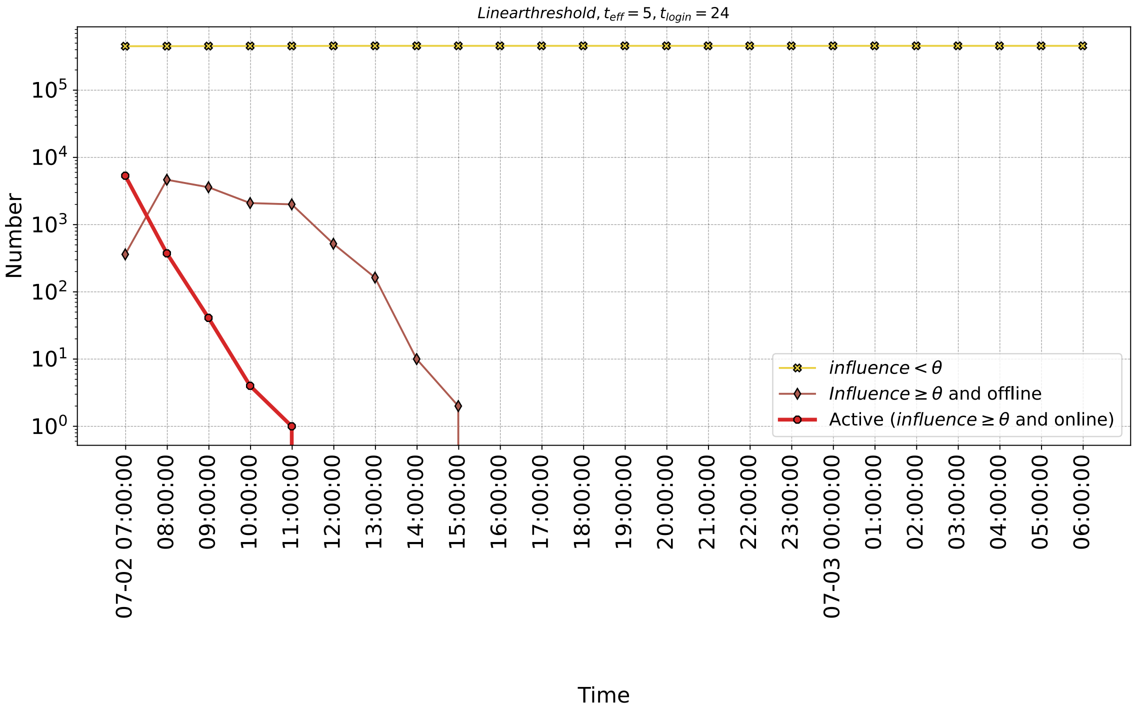

Fig. S5. Distribution of active and online, active and offline, inactive-by-the-lower-threshold, and inactive-by-the-upper-threshold users in the Higgs Boson dataset under the linear threshold model with $T_{\text{effect}} = 5$.

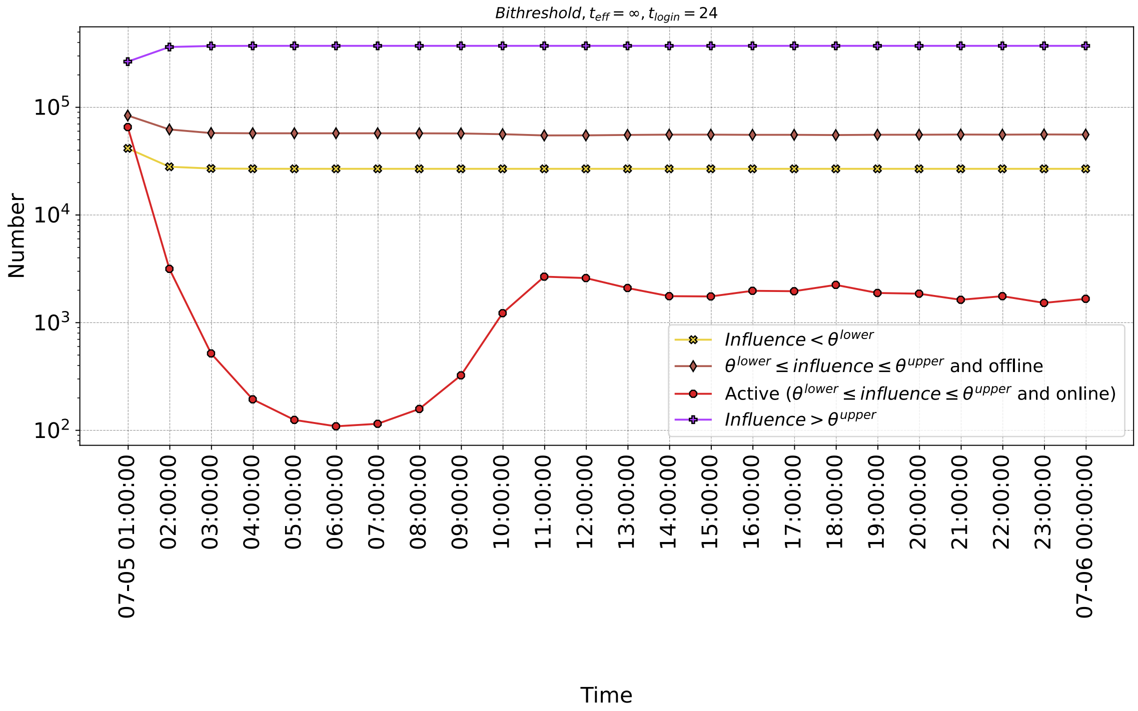

Fig. S6. Distribution of active and online, active and offline, inactive-by-the-lower-threshold, and inactive-by-the-upper-threshold users in the Higgs Boson dataset under the bithreshold model with $T_{\text{effect}} = \infty$.

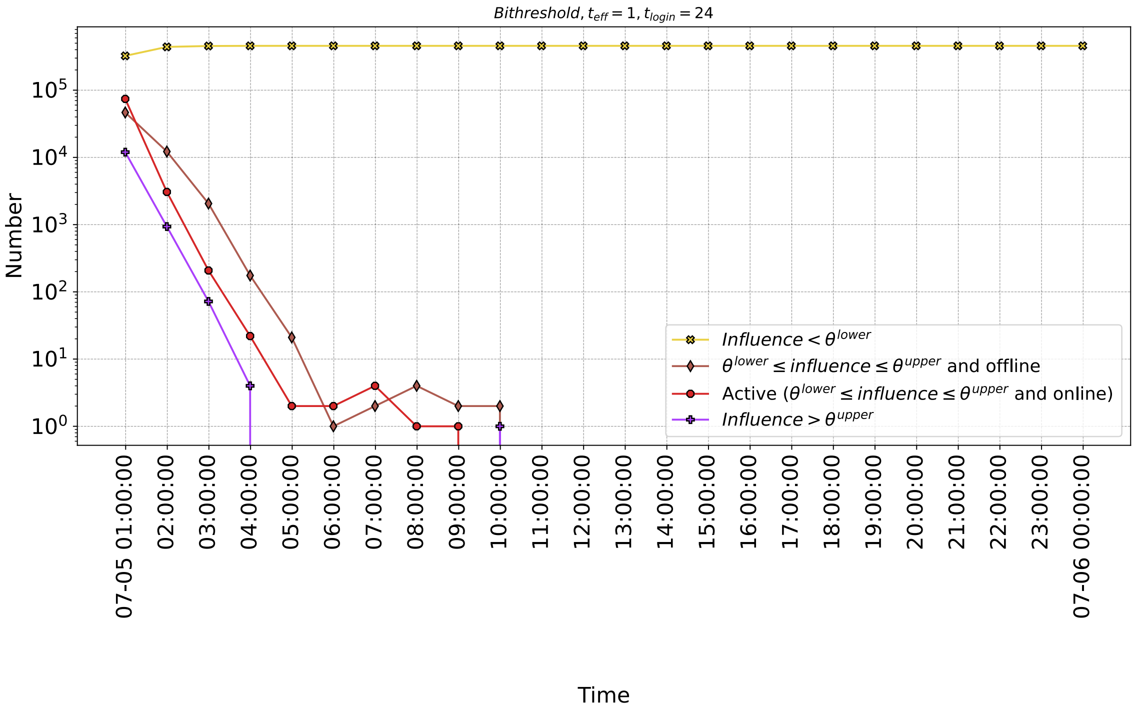

Fig. S7. Distribution of active and online, active and offline, inactive-by-the-lower-threshold, and inactive-by-the-upper-threshold users in the Higgs Boson dataset under the bi-threshold model with $T_{\mathrm{effect}} = 1$.

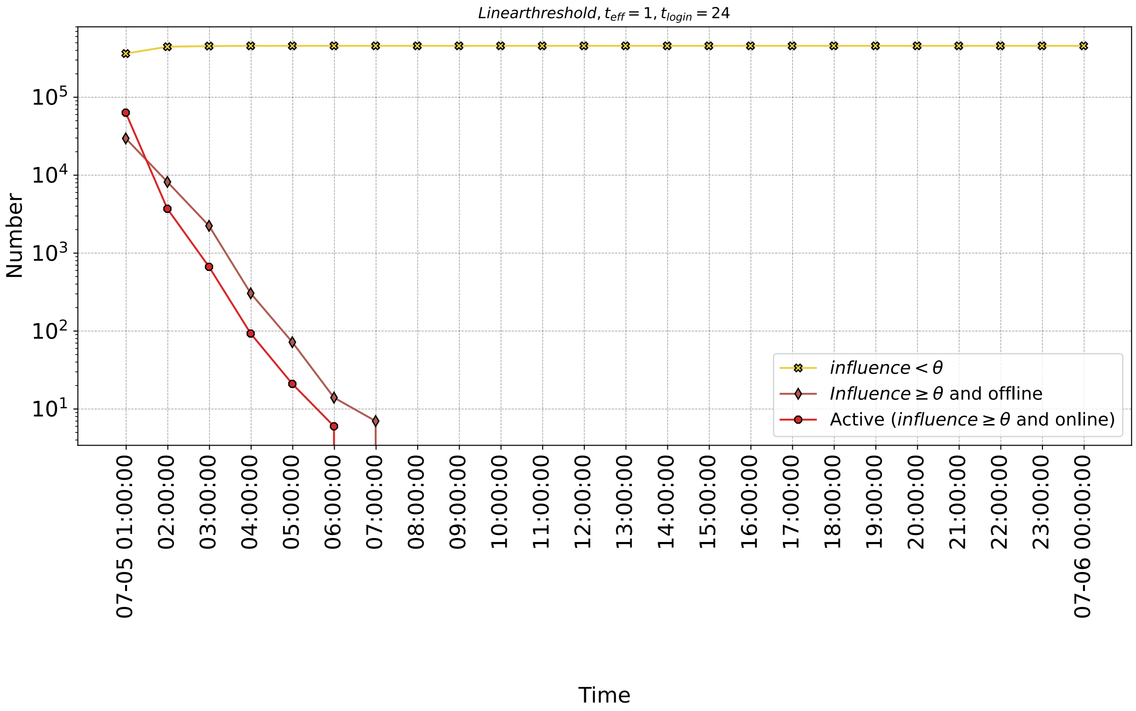

Fig. S8. Distribution of active and online, active and offline, inactive-by-the-lower-threshold, and inactive-by-the-upper-threshold users in the Higgs Boson dataset under the linear threshold model with $T_{\text{effect}} = 1$.

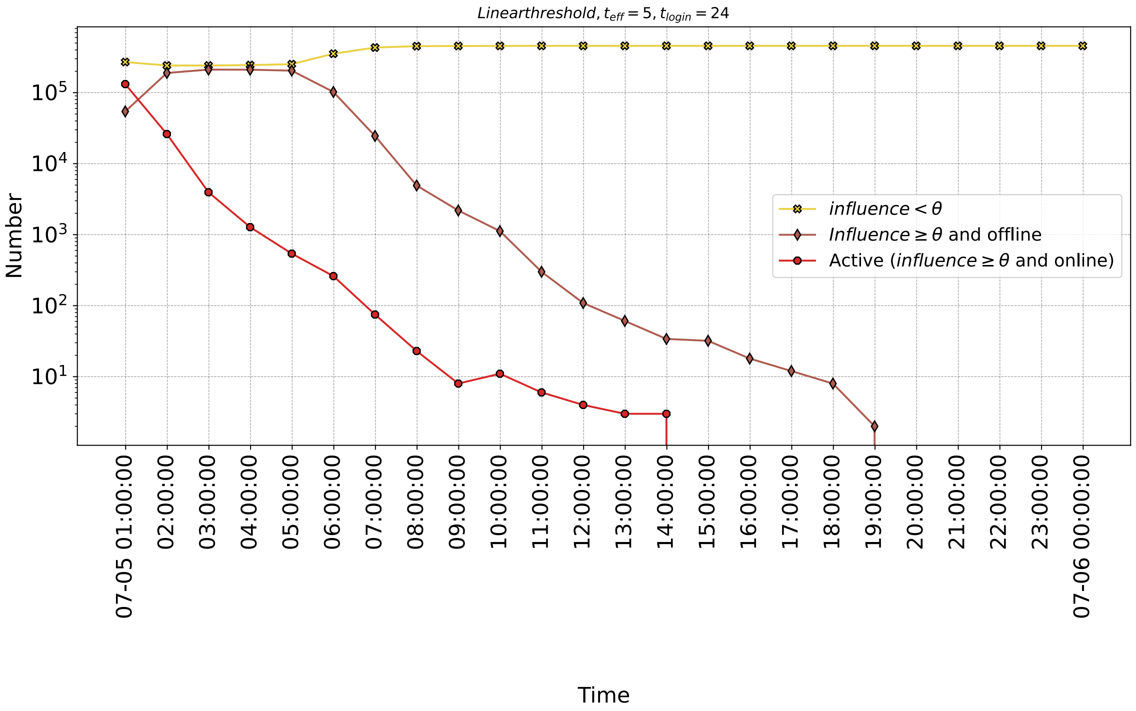

Fig. S9. Distribution of active and online, active and offline, inactive-by-the-lower-threshold, and inactive-by-the-upper-threshold users in the Higgs Boson dataset under the linear threshold model with $T_{\mathrm{effect}} = 5$.

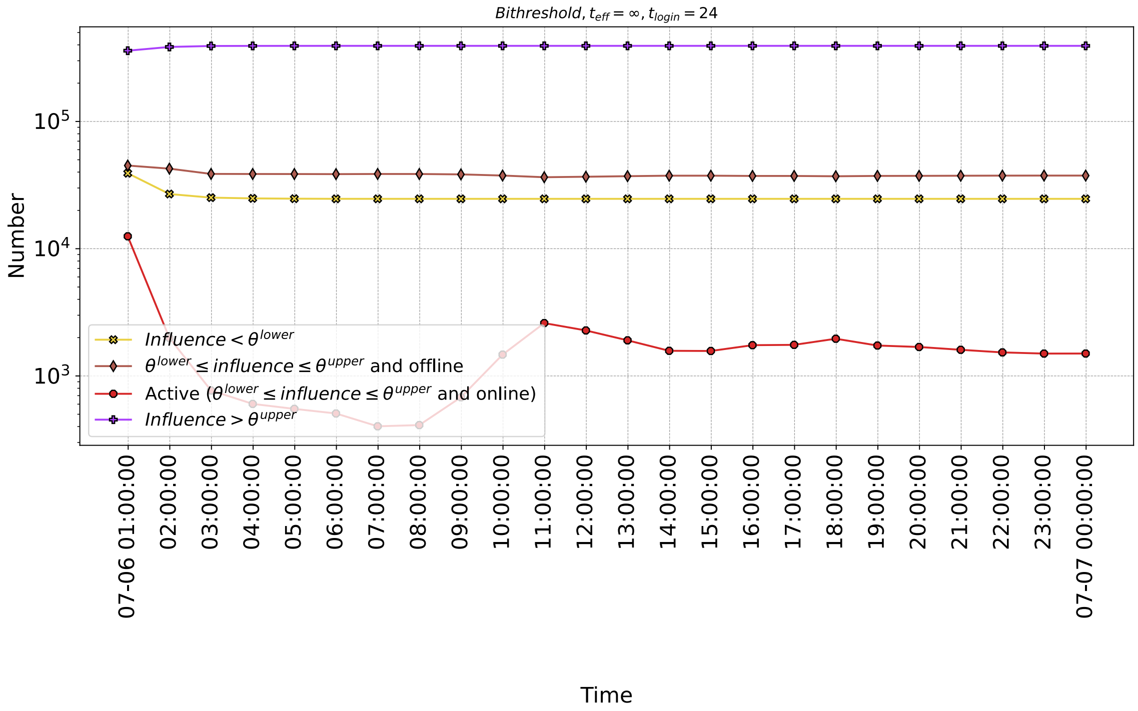

Fig. S10. Distribution of active and online, active and offline, inactive-by-the-lower-threshold, and inactive-by-the-upper-threshold users in the Higgs Boson dataset under the bithreshold model with $T_{\text{effect}} = \infty$.

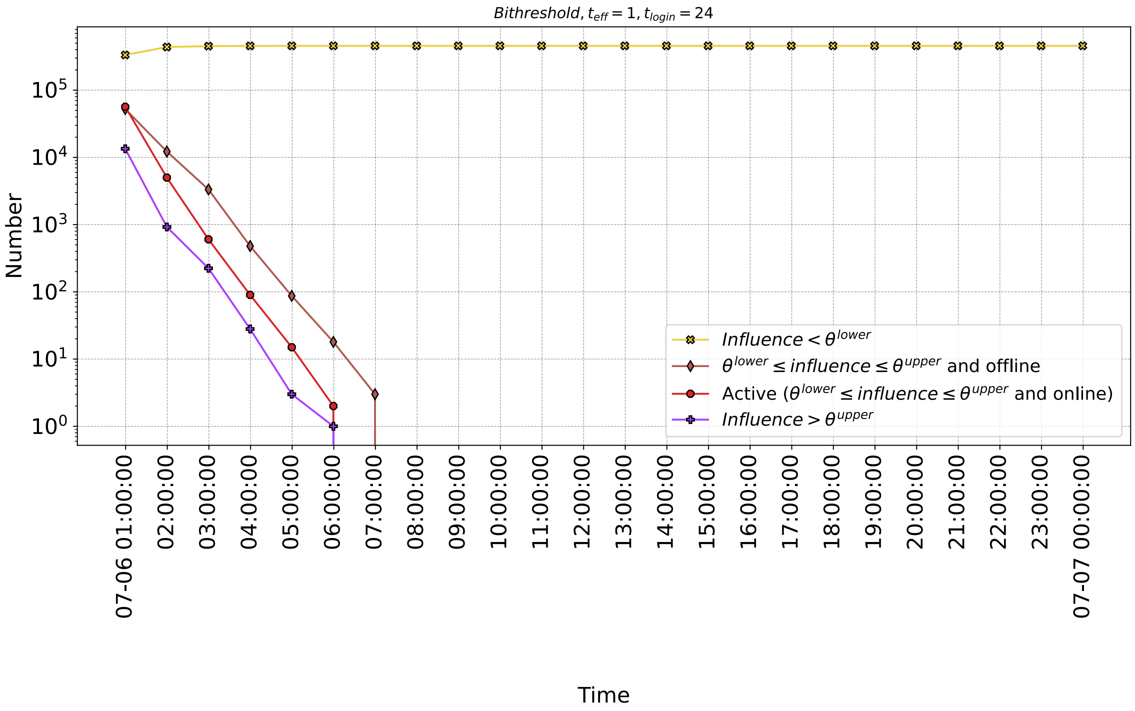

Fig. S11. Distribution of active and online, active and offline, inactive-by-the-lower-threshold, and inactive-by-the-upper-threshold users under the bithreshold model with $T_{\mathrm{effect}} = 1$.

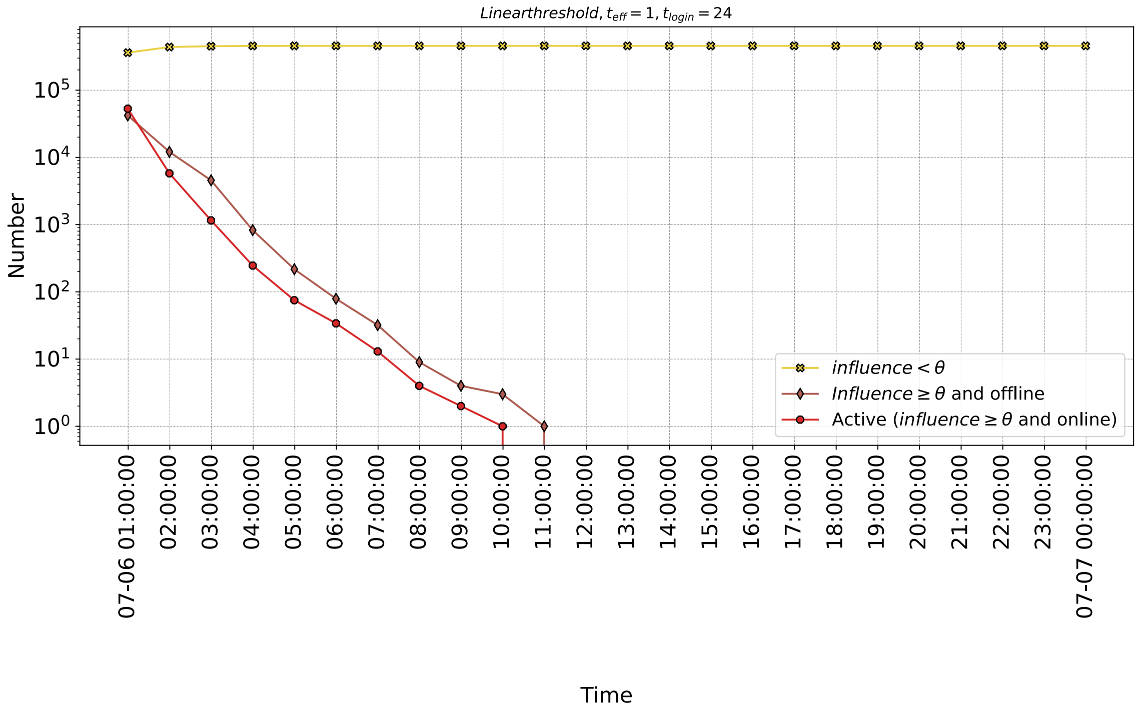

Fig. S12. Distribution of active and online, active and offline, inactive-by-the-lower-threshold, and inactive-by-the-upper-threshold users in the Higgs Boson dataset under the linear threshold model with $T_{\mathrm{effect}} = 1$.

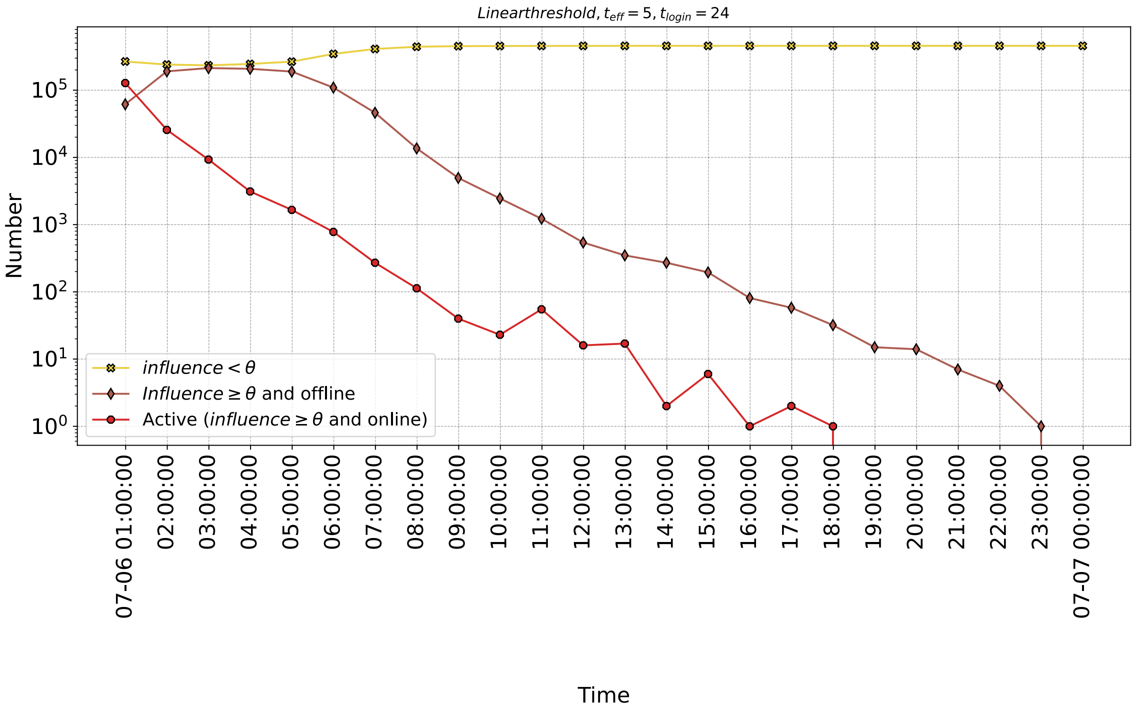

Fig. S13. Distribution of active and online, active and offline, inactive-by-the-lower-threshold, and inactive-by-the-upper-threshold users in the Higgs Boson dataset under the linear threshold model with $T_{\text{effect}} = 5$.

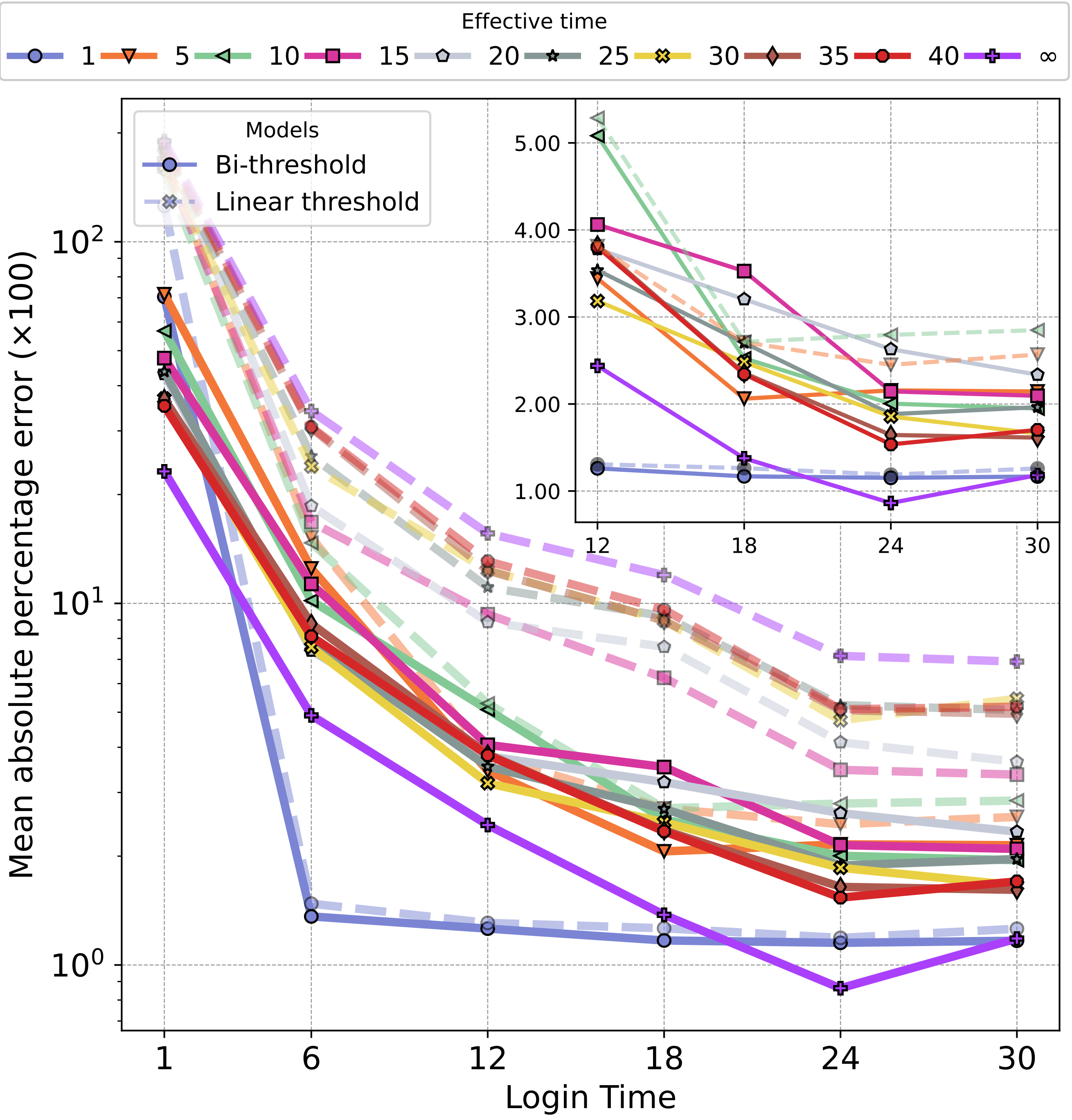

Fig. S14. Performance comparison of the linear threshold and bi-threshold models for the Higgs Boson dataset over different login and effective times. Each point represents the average error over all testing data instances with the specified login and effective time. The dashed and solid lines represent the linear threshold and bi-threshold models respectively. The bi-threshold model with $T_{Login} = 24$ and $T_{Effect} = \infty$ had the lowest error rate.

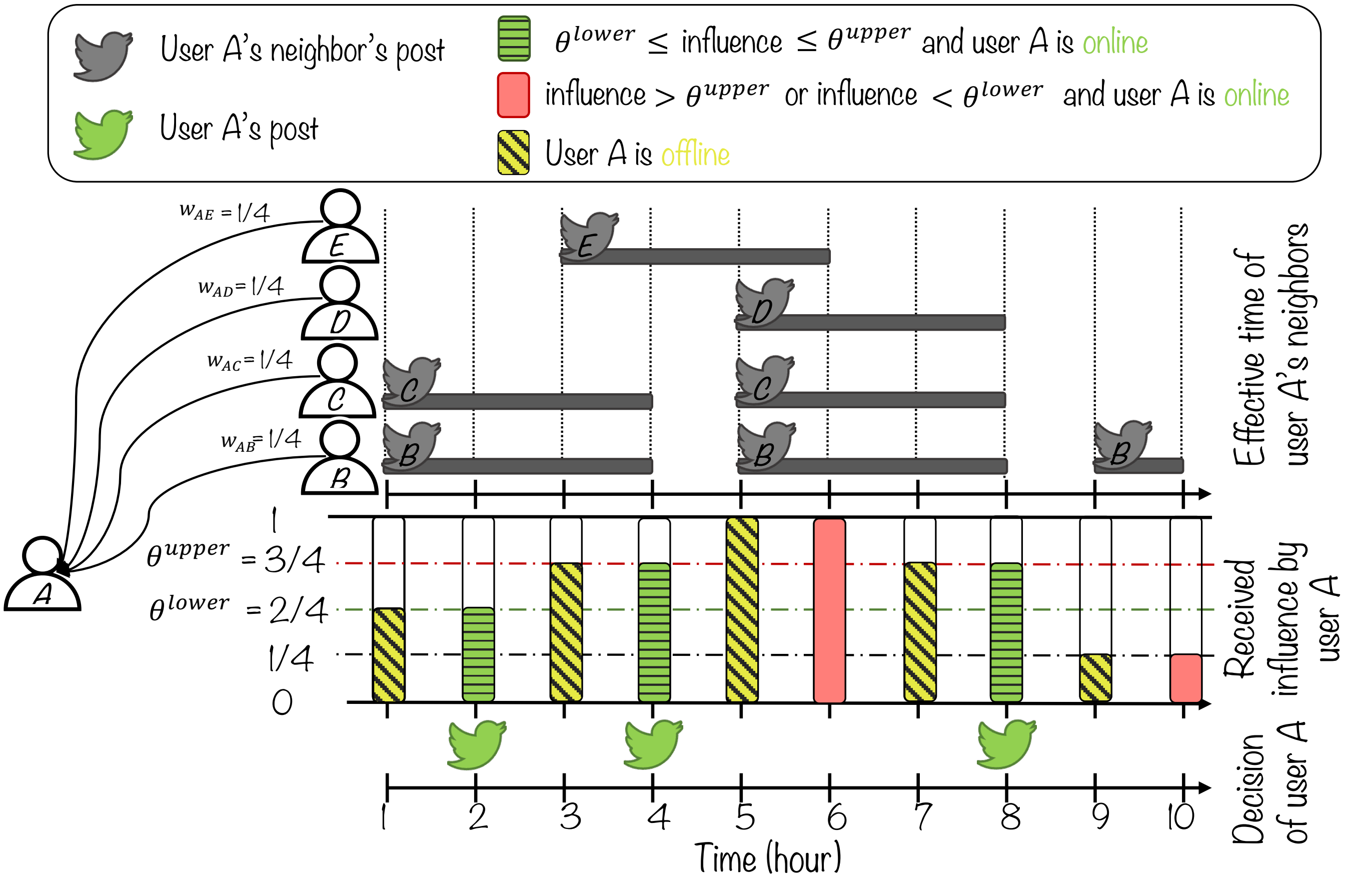

Fig. S15. Illustration of information diffusion on Twitter under the bi-threshold model. Twitter user A follows users B, C, D, and E (i.e., A's network neighbors). Tweets and reactions about a rumor by network neighbors (indicated with the gray birds in the upper timeline) increase the level of social influence received by user A (indicated with the vertical bars in the middle timeline), with each neighbor contributing equally to the social influence (i.e., weights w_{AB}, w_{AC}, w_{AD} , and w_{AE} are all equal to $1/4$ as shown in upper left part of the figure). The impact of a neighbor's tweet is assumed to last for three hours ($T_{effect} = 4$; indicated by gray lines in the upper timeline). For example, at time $t = 1$, neighbors B and C have tweeted, making the received social influence equal to $1/4 + 1/4 = 1/2$ at $t = 1$ and $t = 2$. The influence increases to $3/4$ at $t = 3$ and 4 because of neighbor E's additional tweet. Influences increases again to 1 for $t = 5$ and 6, as B, C, and D tweet, but then decreases to $3/4$ at $t = 7$ as the effect of E's tweet expires. User A is assumed to login every two hours ($T_{login} = 2$, starting from $t = 2$). When logged in ($t = 2, 4, 6, 8,$ and 10), user A will tweet about or react to the rumor if the received social influence is between the lower and upper threshold (green bar and green bird at times $t = 2, 4,$ and 8) or ignore the rumor if the threshold is outside the bounds of the two thresholds (red bar at $t = 6$ and 10).

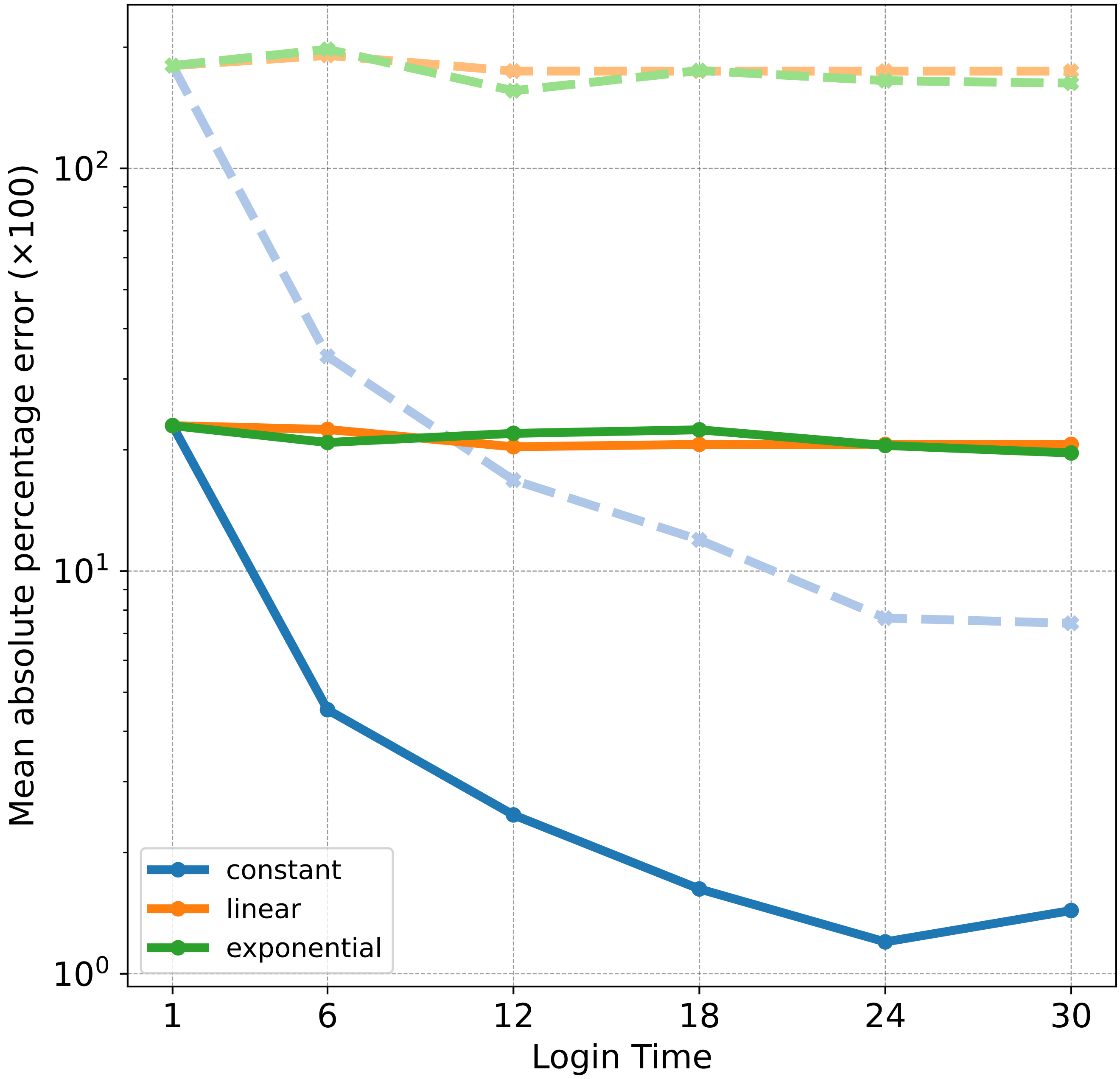

Fig. S16. Performance comparison of the linear threshold and bi-threshold models for the Higgs Boson dataset for different login time method. Each point represents the average mean absolute error over all testing data with login time= ∞

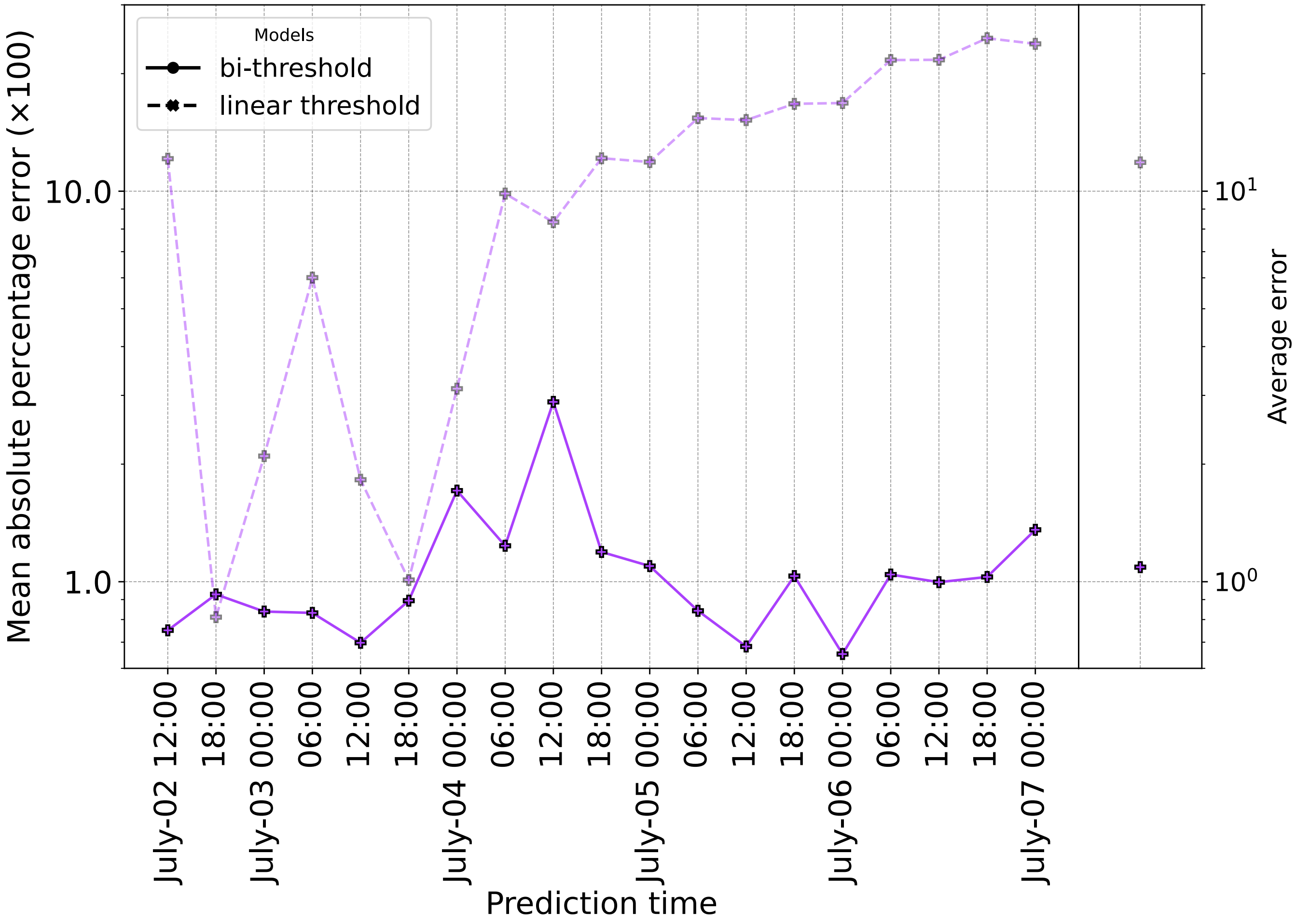

Fig. S17. Performance comparison of the linear threshold and bi-threshold models for the Higgs Boson dataset when the test dataset is not limited to the future 24 hours but extended to the final available time point in the dataset. The same configuration as that of Figure 3 in the manuscript was used for the linear threshold ($T_{\text{login}} = 24, T_{\text{effect}} = \infty$) and bi-threshold models ($T_{\text{login}} = 24, T_{\text{effect}} = \infty$). The MAPE of the bi-threshold model (109%) is less than that of the linear threshold model (1,185%).

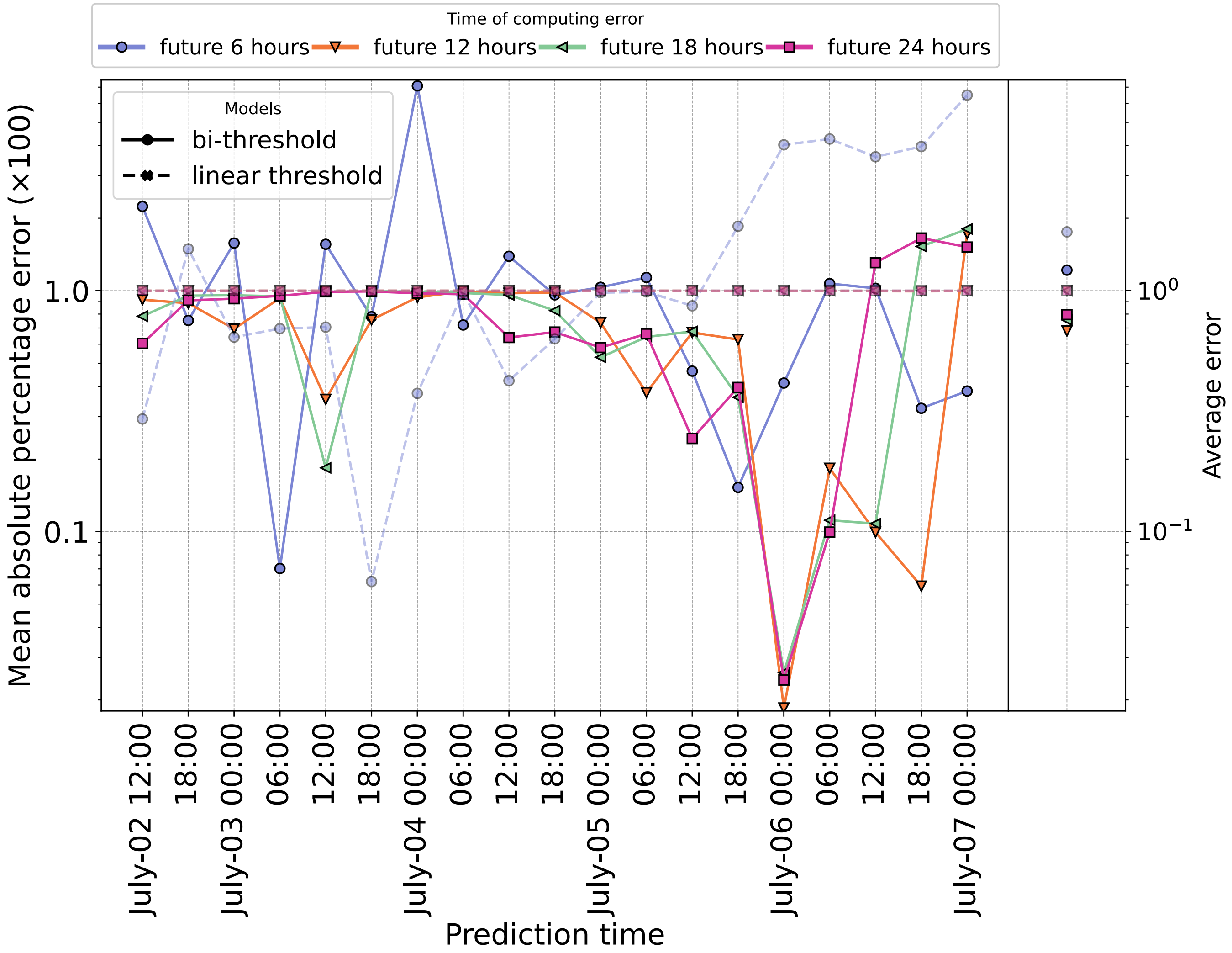

Fig. S18. Performance comparison of the linear threshold and bi-threshold models for the Higgs Boson dataset for future 6, 12, 18, and 24 hours separately.

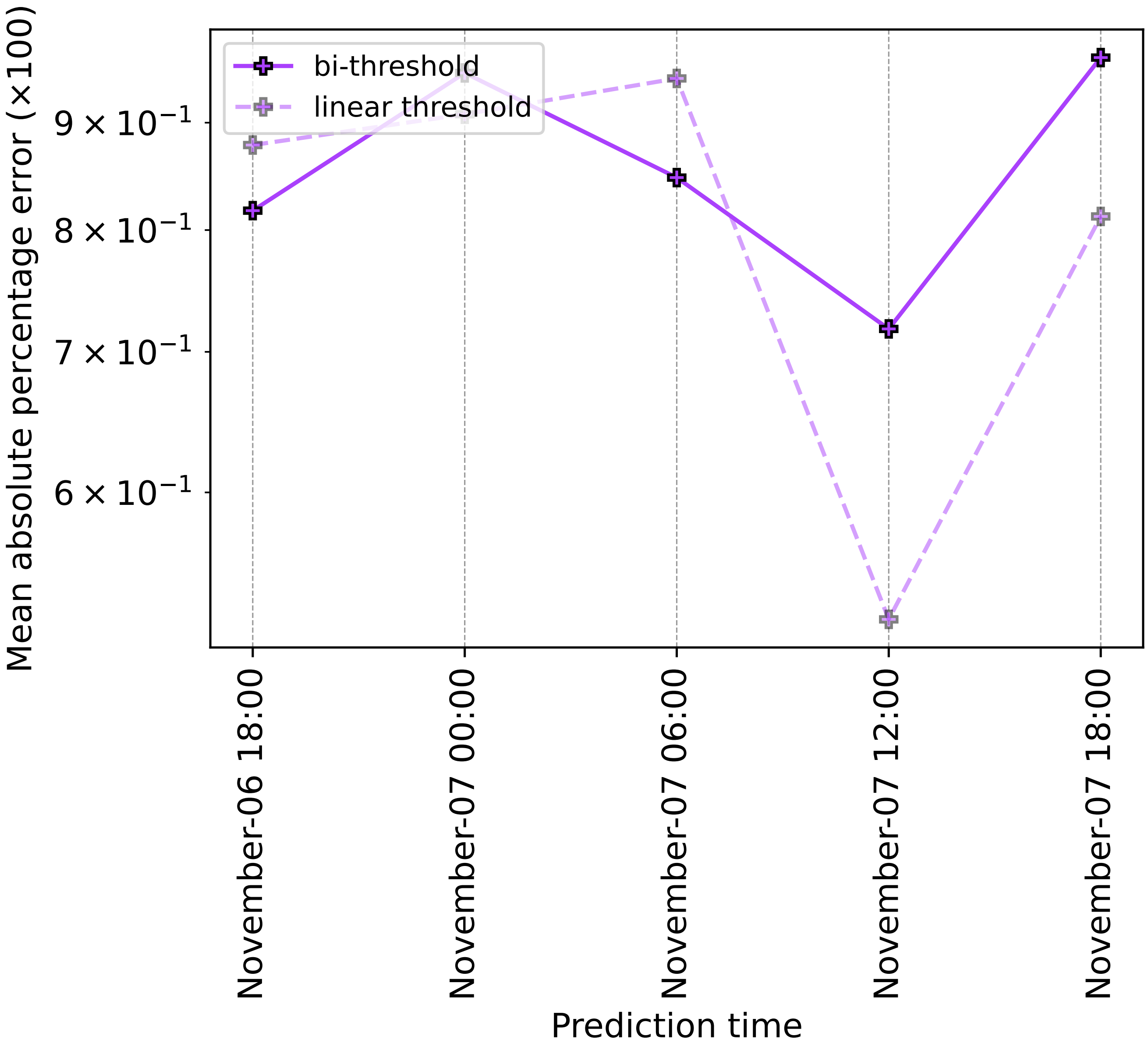

Fig. S19. Mel-cup dataset. Performance comparison of the linear threshold and bi-threshold models without an effective time and for a login frequency of 24 hours. The setup of this figure is the same as that of Figure 3 in the main article.

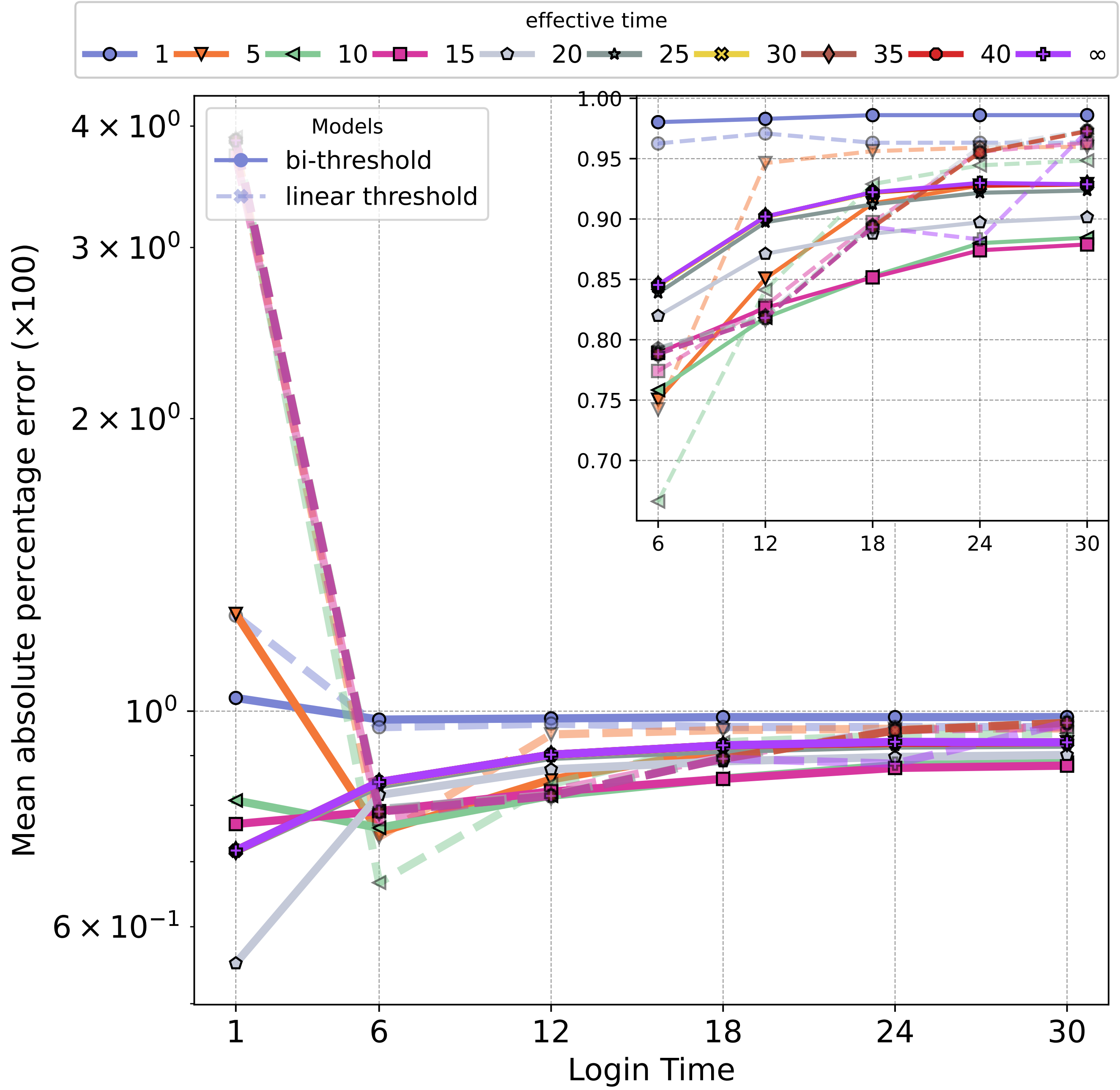

Fig. S20. Mel-cup dataset. Performance comparison of the linear threshold and bi-threshold models for different T_{login} and T_{effect}. The setup of this figure is the same as that of Figure S14.

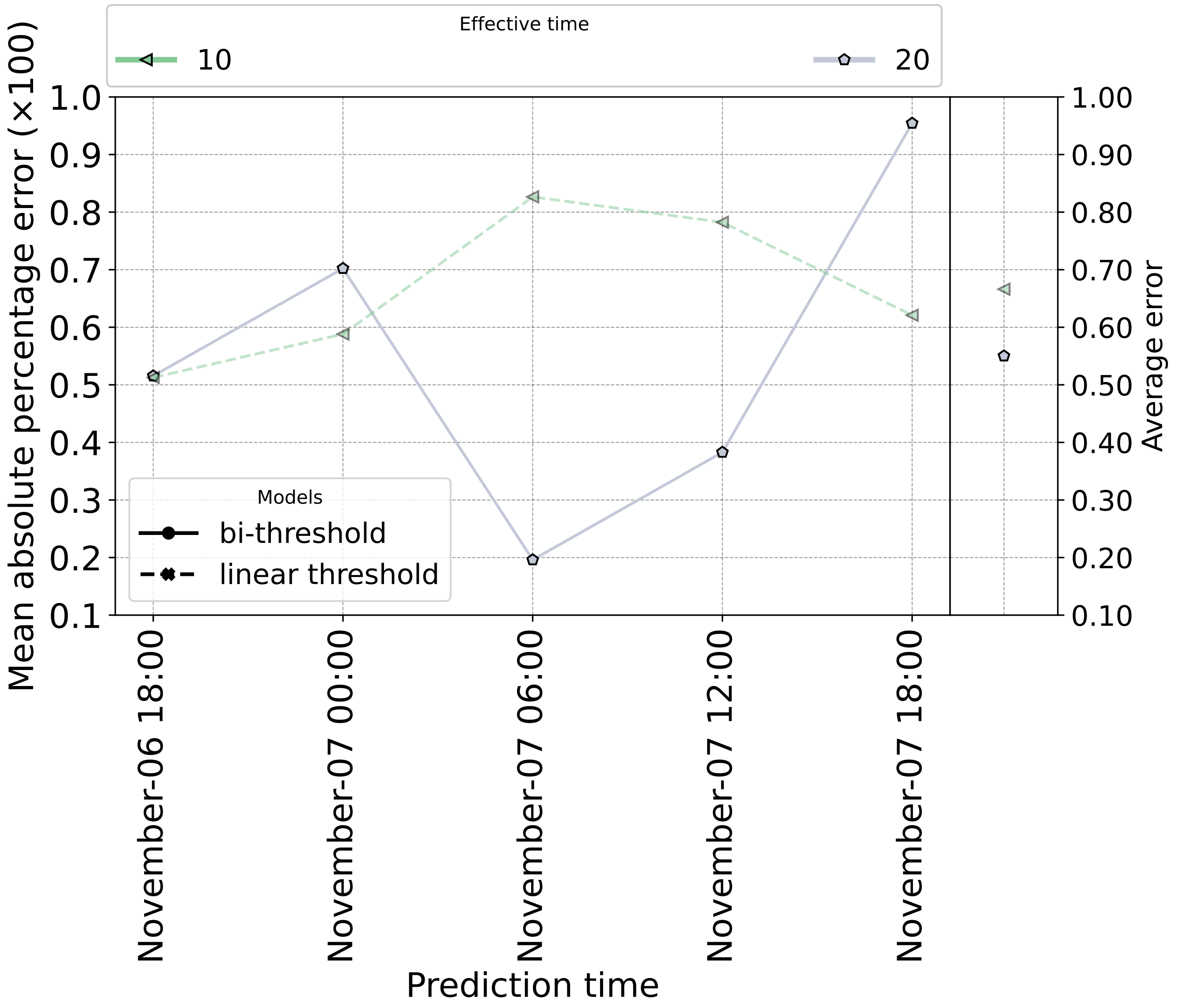

Fig. S21. Mel-cup dataset. Performance comparison of the best linear threshold ($T_{\mathrm{login}} = 6, T_{\mathrm{effect}} = 10$) and best bi-threshold ($T_{\mathrm{login}} = 1, T_{\mathrm{effect}} = 20$).

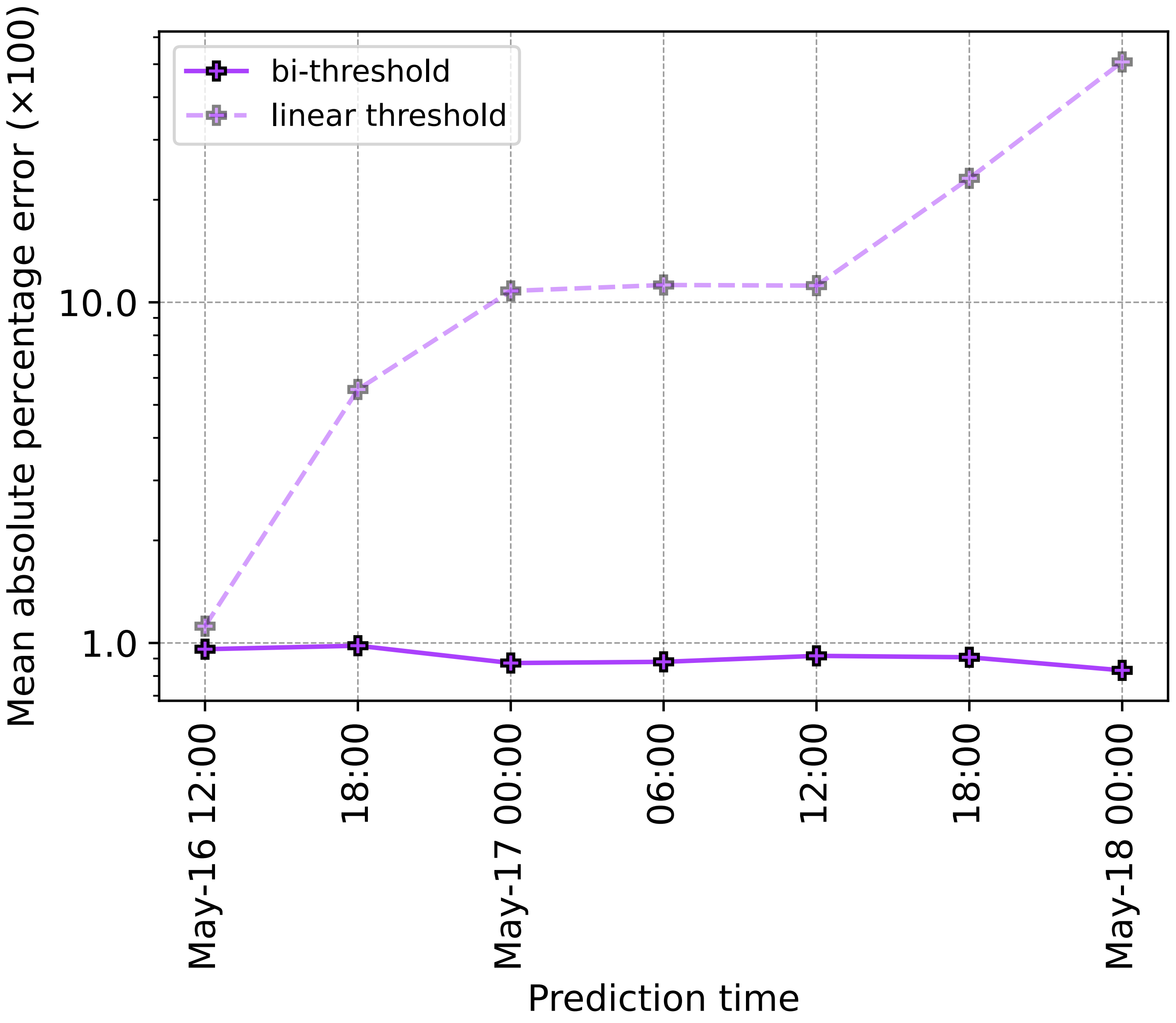

Fig. S22. Weibo-dataset. Performance comparison of the linear threshold and bi-threshold models without an effective time and for a login frequency of 24 hours. The setup of this figure is the same as that of Figure 3 in the main article.

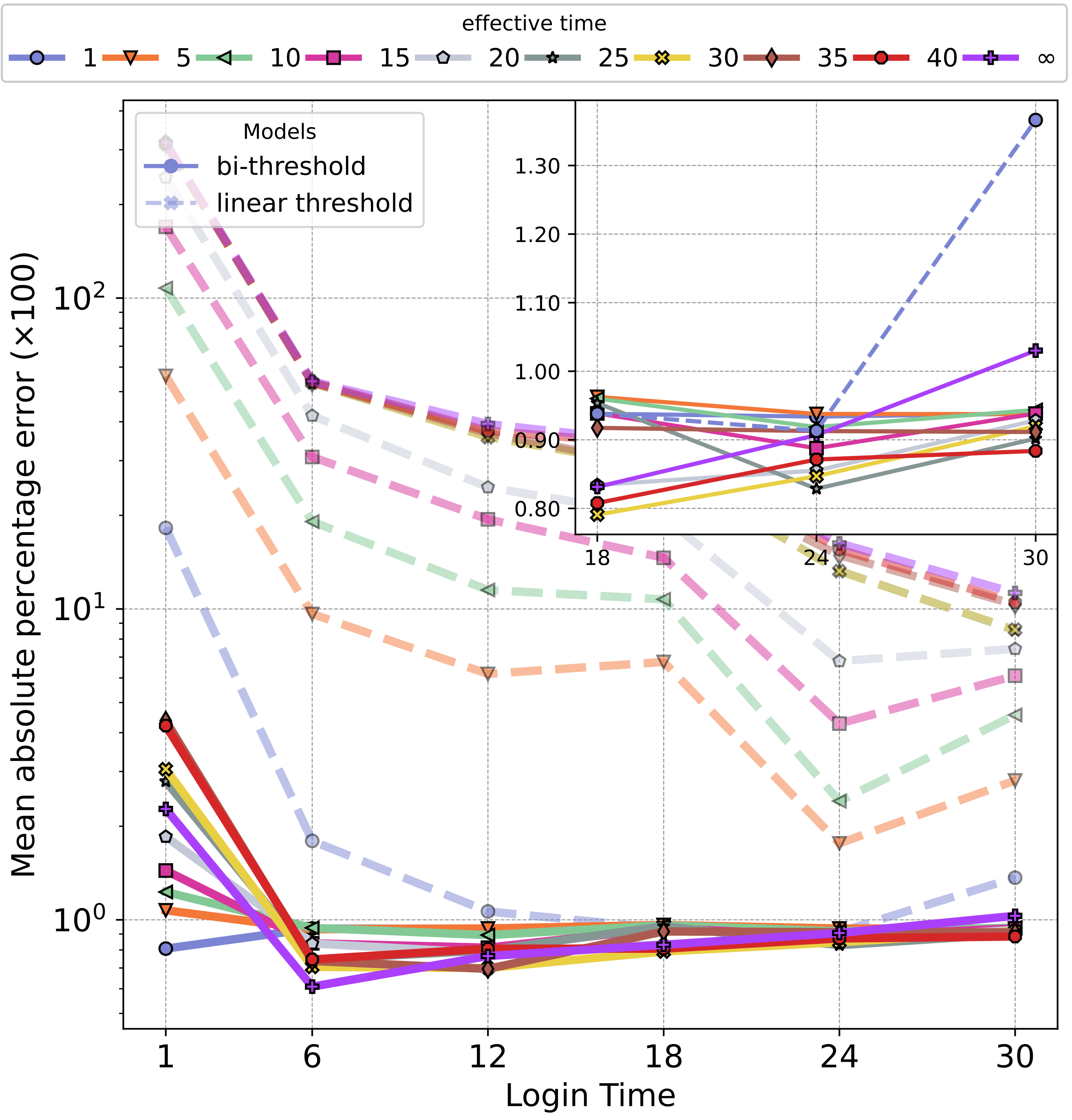

Fig. S23. Weibo-dataset. Performance comparison of the linear threshold and bi-threshold models for different T_{login} and T_{effect}. The setup of this figure is the same as that of Figure S14.

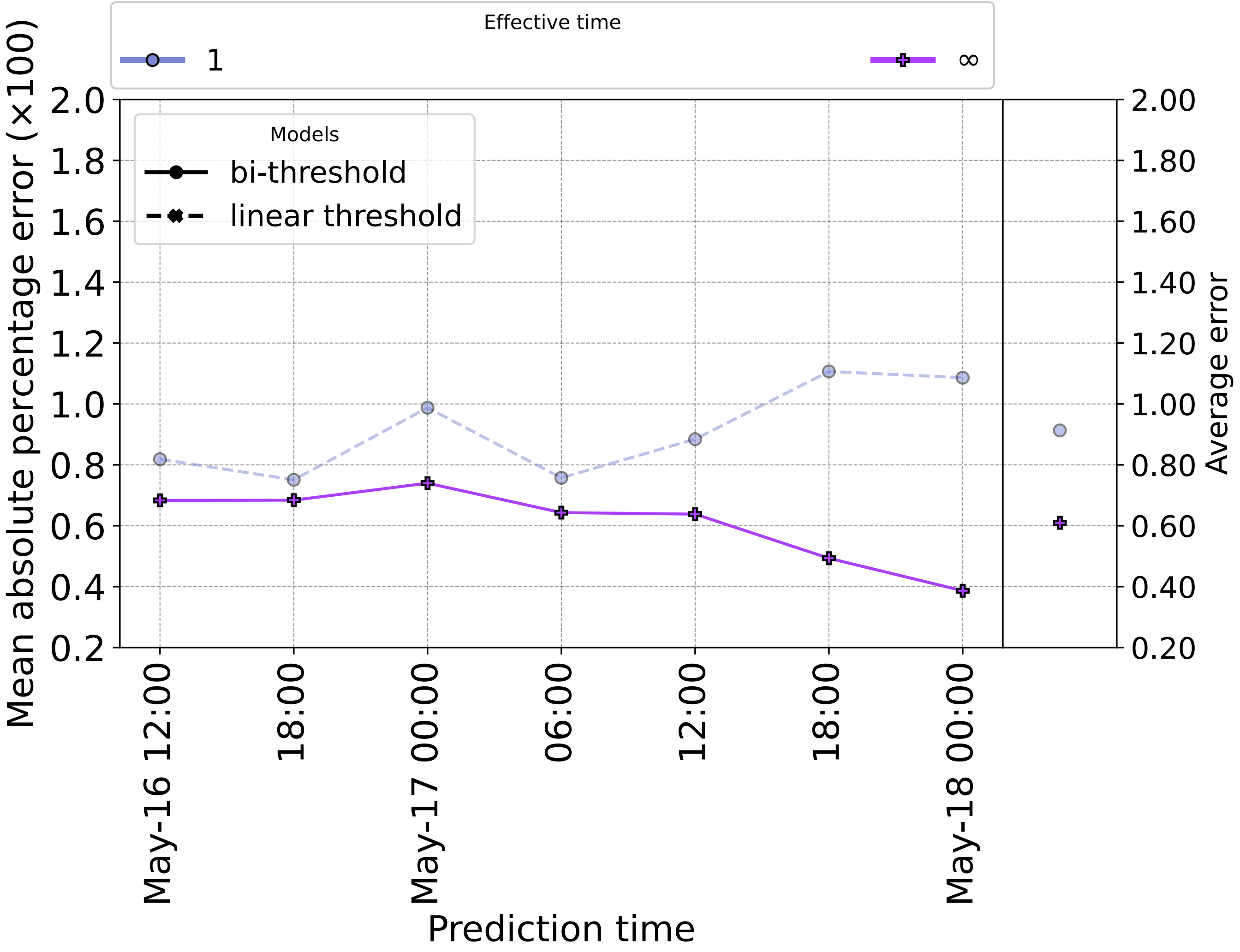

Fig. S24. Weibo-dataset. Performance comparison of the best linear threshold ($T_{\text{login}} = 24, T_{\text{effect}} = 1$) and best bi-threshold ($T_{\text{login}} = 6, T_{\text{effect}} = \infty$).

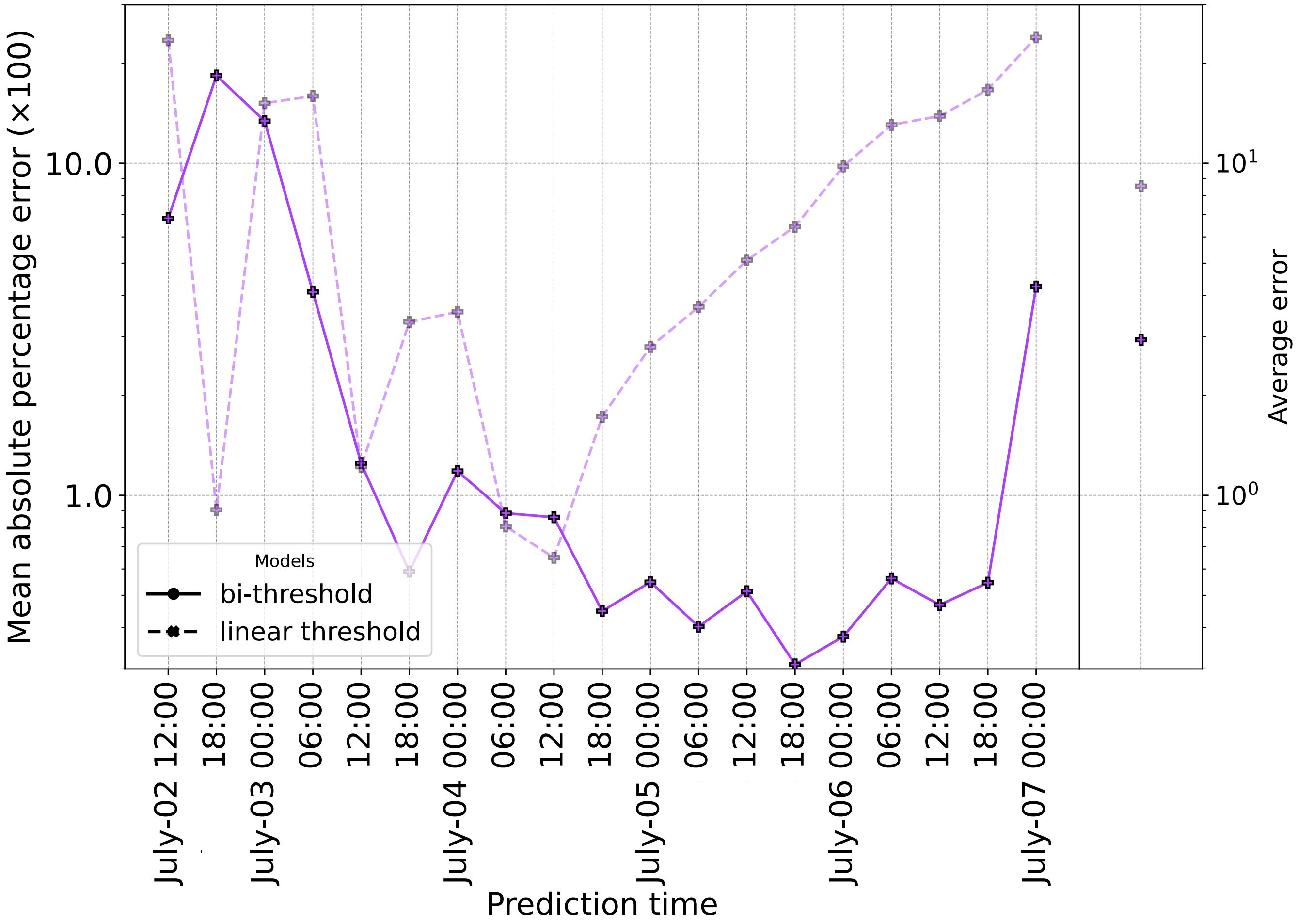

Fig. S25. Higgs Boson dataset robustness check. Performance comparison of the linear threshold and bi-threshold models using a consistent, preceding 24-hour training window. The analysis uses the same model configuration as in Figure 3 of the manuscript ($T_{\mathrm{login}} = 24, T_{\mathrm{effect}} = \infty$). This analysis is to show that the changing size of the training window does not affect the results. The MAPE of the bi-threshold model (294%) is less than that of the linear threshold model (853%)

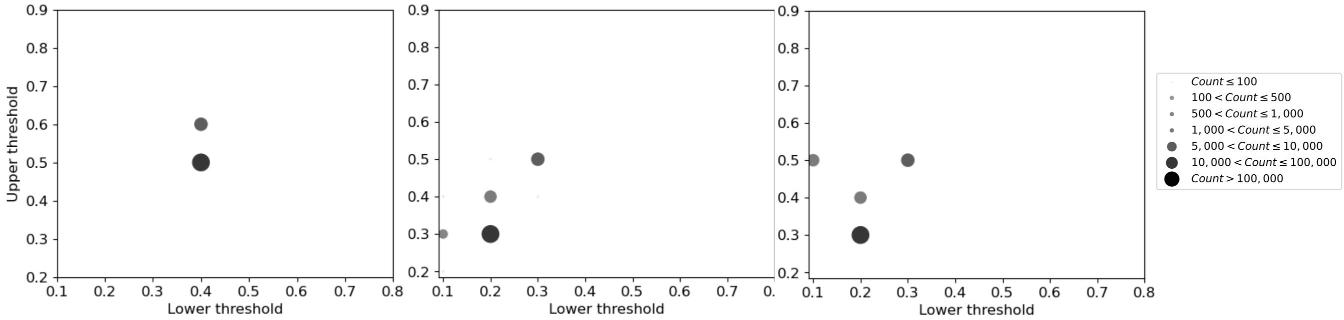

a) The beginning of the spread

b) The peak of the spread

c) The end of the spread

Fig. S26. Distribution of threshold estimates across different stages of the weibo news spread. The distribution of the estimated lower and upper thresholds for the bi-threshold model with $T_{\text{effect}} = \infty, T_{\text{login}} = 24$ are provided for the time intervals **a)** May-16 12:00 to May-16 18:00, as the beginning of the news spread, **b)** May-17 00:00 to May-17 12:00, as the peak of spread, and **c)** after May-17 18:00, as the final stage of the spread. The size of the circles is proportional to the frequency of individuals with the specified lower and upper thresholds.

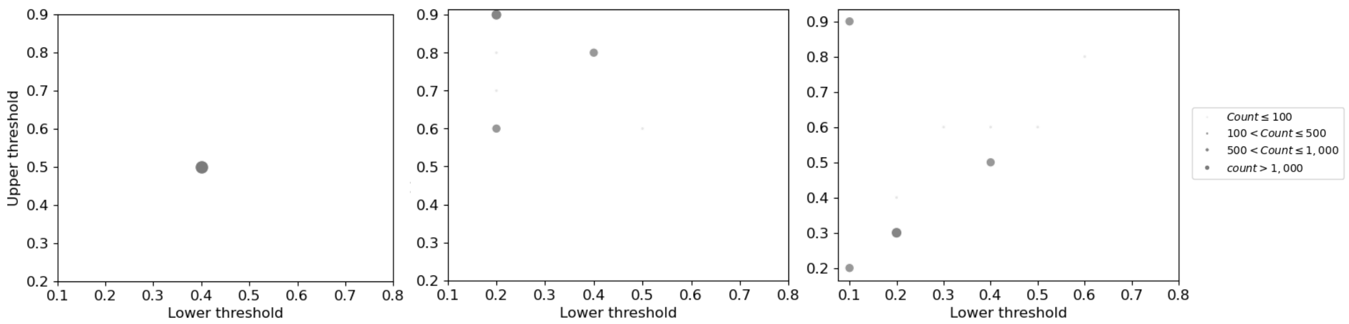

a) The beginning of the spread b) The peak of the spread c) The end of the spread

Fig. S27. Distribution of threshold estimates across different stages of the Mel-cup news spread. The distribution of the estimated lower and upper thresholds for the bi-threshold model with $T_{\text{effect}} = \infty, T_{\text{login}} = 24$ are provided for the time intervals **a)** November-6 18:00 to November-7 00:00, as the beginning of the news spread, **b)** November-7 06:00 to November-7 12:00, as the peak of spread, and **c)** after November-7 18:00, as the final stage of the spread. The size of the circles is proportional to the frequency of individuals with the specified lower and upper thresholds.

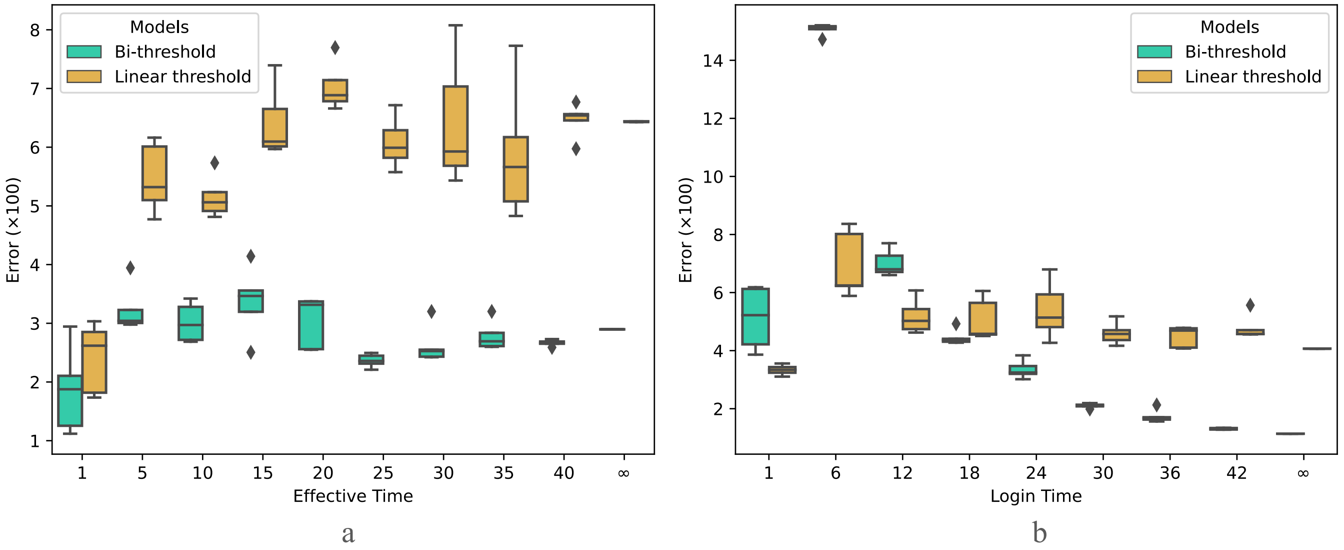

Fig. S28. Higgs Boson dataset. The performance of the linear threshold and bi-threshold models for a heterogeneous population where individuals have different values of T_{login} and T_{effect}. a) The default value of $T_{\text{effect}} = \infty$ as in Fig. 3 in the manuscript was assigned to all users. The users' T_{login} values followed a Poisson distribution with a random assignment of the values to the users. Ten different populations were tested for the Poisson means of $1, 5, 10, 15, 20, 25, 30, 35, 40, \infty$ as indicated by the x-axis. For each population, we repeated five times the same procedure as in that of Fig. 3 in the manuscript, i.e., obtaining the thresholds of the individuals on the training dataset and testing their performance on the testing dataset. The mean absolute percentage error of the five experiments were averaged and reported on the y-axis. For each value of the x-axis (T_{effect}), the left (green) box represents the box-plot of the bi-threshold and the right (yellow) is for the linear threshold model. **b)** The same as part a), with the difference that here $T_{\text{effect}} = \infty$ and T_{login} follows the Poisson distribution.

3. The Mel-cup dataset

The processed dataset contains all tweets that included one of the following terms: "Melbourne Cup", "#MelCup", and "DerbyDay, from 13:00:00 6 November 2017 to 04:00:00 8 November 2017. We focus attention on the "tweet" (including "similar content" tweet), "retweet," "reply," and "quote," social reactions, and exclude "likes" from the analysis. The dataset did not include the "mentions". The resulting dataset captures the activity of 1,437 Twitter users, who had 70,806 following relationship edges among them. As in the Higgs Boson dataset, we transformed the raw data into an hourly dataset, creating a binary indicator of each user i's decision ($y_i(t)$) to engage at time t, which equals 1 if the user recorded a social reaction to any message with a relevant hashtag at least once during that hour and 0 otherwise. The time interval from 13:00:00 6 November 2017 to 18:00:00 6 November 2017 was used only for training, and the time interval from 18:00:00 7 November 2017 to 04:00:00 8 November 2017 was used only for testing the models.

4. The Weibo dataset

The dataset contains all messages that included one of the following terms in Chinese: "vaccine," "vaccination," "adenovirus vector," "inactivation," "clinical trail, " "Phase III trail," "immune," "antibody," "mutant virus," "herd immunity," "novel coronavirus," "corona virus," "Covid," "novel corona pneumonia," "Wuhan's unknown pneumonia," "pneumonia of unknown cause," "nucleic acid testing," "Wuhan pneumonia," "human-to-human transmission," and the following in English: "COVID-19," "COVID," "COVID19," "SARS," "SARS-2," "SARS-CoV-2," "nCoV," "2019-nCoV." The time interval was from 00:00:00 29 April 2021 to 04:00:00 18 May 2021. It captures the activity of 1,052,896 users, who have 3,841,030 following relationship edges among them. We focus attention on the "post," "re-post," and "comment" social reactions. As in the Higgs Boson dataset, we transformed the raw data into an hourly dataset, creating a binary indicator of each user i's decision ($y_i(t)$) to engage at time t, which equals 1 if the user recorded a social reaction to any message with a relevant term at least once during that hour and 0 otherwise. Due to the activity sparseness in this dataset, the interval from the beginning of the dataset up to noon 16 May 2021 was used for the training. When less data was used for the training, the models almost learned nothing and poorly estimated the thresholds.
